# Antibody blockade of CLEC12A delays EAE onset and attenuates disease severity by impairing myeloid cell CNS infiltration and restoring positive immunity

**DOI:** 10.1038/s41598-017-03027-x

**Published:** 2017-06-02

**Authors:** Divya Sagar, Narendra P. Singh, Rashida Ginwala, Xiaofang Huang, Ramila Philip, Mitzi Nagarkatti, Prakash Nagarkatti, Konstantin Neumann, Jürgen Ruland, Allison M. Andrews, Servio H. Ramirez, Zafar K. Khan, Pooja Jain

**Affiliations:** 1 Department of Microbiology and Immunology, Drexel University College of Medicine, Philadelphia, PA USA; 2 Department of Pathology, Microbiology and Immunology, School of Medicine, University of South Carolina, Columbia, SC USA; 3 William Jennings Bryan Dorn VA Medical Center, Columbia, SC USA; 4 Immunotope Inc., Pennsylvania Biotechnology Center, Doylestown, PA USA; 5 Institut für Klinische Chemie und Pathobiochemie, Klinikum rechts der Isar, Technische Universität München, Munich, Germany; 6 Department of Pathology and Laboratory Medicine, Lewis Katz School of Medicine, Temple University, Philadelphia, PA USA

## Abstract

The mechanism of dendritic cells (DCs) recruitment across the blood brain barrier (BBB) during neuroinflammation has been the least explored amongst all leukocytes. For cells of myeloid origin, while integrins function at the level of adhesion, the importance of lectins remains unknown. Here, we identified functions of one C-type lectin receptor, CLEC12A, in facilitating DC binding and transmigration across the BBB in response to CCL2 chemotaxis. To test function of CLEC12A in an animal model of multiple sclerosis (MS), we administered blocking antibody to CLEC12A that significantly ameliorated disease scores in MOG_35–55_-induced progressive, as well as PLP_138–151_-induced relapsing-remitting experimental autoimmune encephalomyelitis (EAE) mice. The decline in both progression and relapse of EAE occurred as a result of reduced demyelination and myeloid cell infiltration into the CNS tissue. DC numbers were restored in the spleen of C57BL/6 and peripheral blood of SJL/J mice along with a decreased TH17 phenotype within CD4^+^ T-cells. The effects of CLEC12A blocking were further validated using CLEC12A knockout (KO) animals wherein EAE disease induction was delayed and reduced disease severity was observed. These studies reveal the utility of a DC-specific mechanism in designing new therapeutics for MS.

## Introduction

The central nervous system (CNS) is structured to be an immune-privileged site to remain protected from detrimental insults that can result in immune-mediated inflammation. Focal demyelinated lesions and transected axons in neuroinflammatory disease such as multiple sclerosis (MS) is believed to be mediated by infiltrating inflammatory cells, including CD4^+^ and CD8^+^ T-cells, B cells, and APCs such as dendritic cells (DCs) and macrophages^1–3^. In a recent study^3^, onset of experimental autoimmune encephalomyelitis (EAE), the mouse model for MS, was shown to coincide with a sudden spike in the number of infiltrating DCs and macrophages in the CNS, the majority of which contained myelin antigen after migration into the CNS.

Amongst the current MS treatments targeting leukocyte infiltration across the blood brain barrier (BBB), natalizumab, a monoclonal antibody against the α-chain of VLA-4^4^, sometimes leads to progressive multifocal leukoencephalopathy^5, 6^ arising out of immune suppression^7–10^ and reactivation of the John Cunningham virus within the CNS of certain patients. In the light of these concerns, our approach to find a target to block myeloid cell migration to evade complete immune suppression is novel.

Studies of EAE have long substantiated the pathogenic role of macrophages^11–13^, but a similar role for DCs has always been postulated^14–19^. Thus far, there has been no attempt to develop a clinically viable target to impede the migration of DCs and other myeloid cells so as to prevent potential reactivation of encephalitogenic lymphocytes. We established the role of the chemokine CCL2 in the trafficking of DCs across the BBB and showed for the first time the real-time trafficking of DCs in the inflamed spinal cord of animals afflicted with EAE^2, 20^. However, the mechanisms (reviewed previously^21^) of how circulating DCs access the CNS remain to be investigated. Therefore, we focused our efforts on understanding C-type lectin receptors (CLRs) found on cells of myeloid origin and have dual roles in cell-adhesion and pathogen-recognition^22^, for their potential role influencing cellular trafficking across the BBB.

Our studies revealed CLEC12A, a Src homology region 2 domain-containing phosphatase 1 and 2 (SHP-1 and -2)-associated receptor involved in inhibitory signaling^23^ as a key molecule to target on immature DCs trafficking to the CNS prior to becoming activated within the CNS upon encountering myelin antigens. Binding of the CLEC12A receptor to the endothelium was demonstrated to be important for monocyte-derived dendritic cells (MDDC)s that are important in development of inflammatory and autoimmune disease^24^ and myeloid DCs (mDCs). In EAE mice, administration of blocking antibody against CLEC12A receptor achieved significant disease attenuation in both progressive and relapsing-remitting EAE models. Reduction in disease severity in antibody-treated mice correlated with reduction in DC accumulation into the CNS tissues, demyelination as well as the TH17 phenotype within CD4^+^ T-cells. Our results were further validated in the CLEC12A^−/−^ animals wherein mice showed a delayed-onset of disease and significant reduction in disease severity. This study opens up the prospect of selectively regulating DC entry into the CNS using antibody treatment as a new alternative against disease pathogenesis and propagation in multiple sclerosis and other inflammatory/autoimmune diseases.

## Results

### Differential surface expression of lectins on different DC subsets

**Figure 1:**
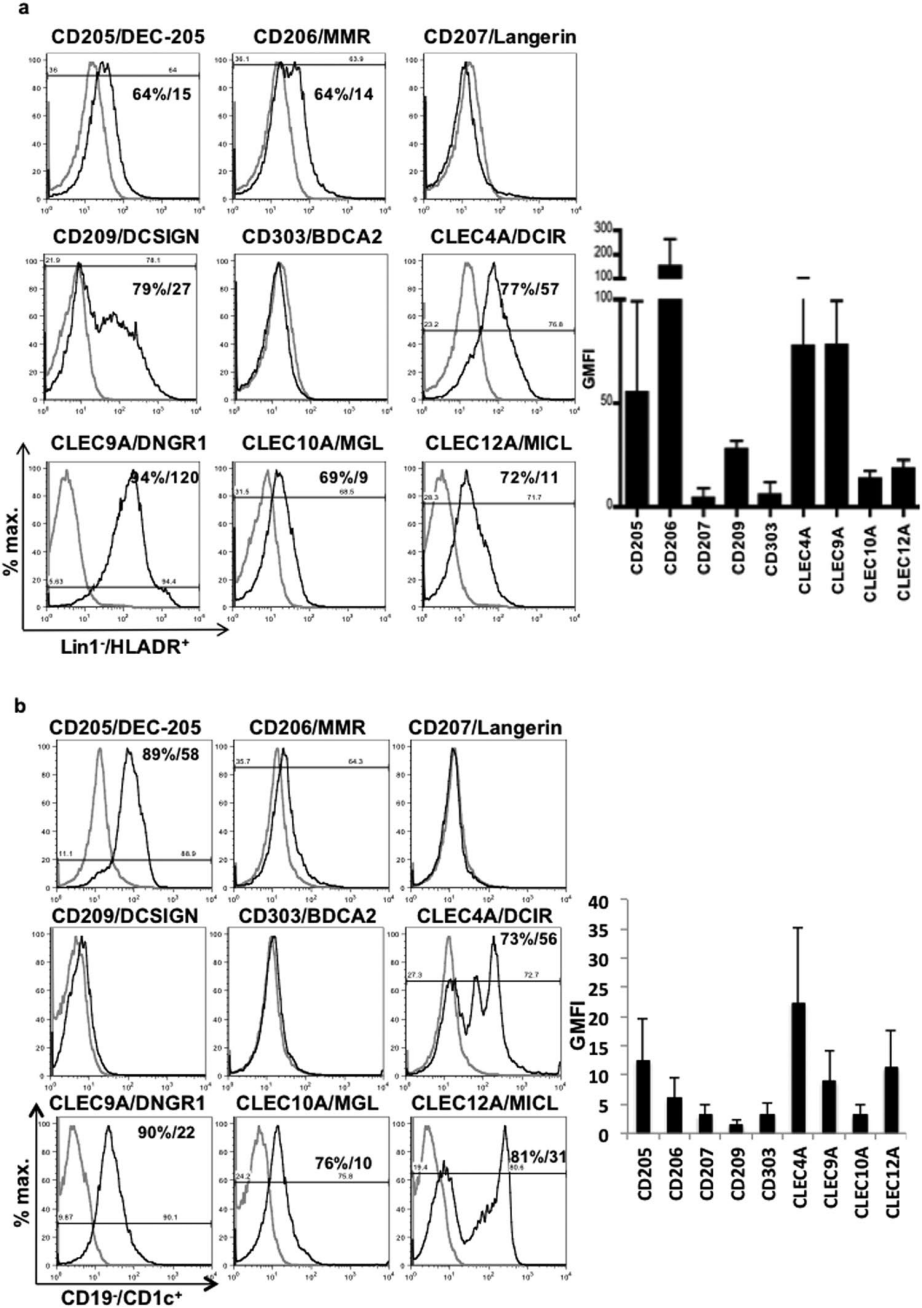
Lectin expression profile on human dendritic cell subsets. PBMCs isolated from individual donors were used to obtain MDDCs and mDCs. Representative histograms of (**a**) lin1^−^/HLADR^+^ MDDCs and (**b**) CD19^−^/Cd1c^+^ mDCs showing individual CLR expression profile with expression levels (black) gated on isotype controls (gray). Comparative geometric mean fluorescence intensities (GMFI) are displayed alongside histograms as bar graphs. Each histogram is representative of three individual donors. Bar graphs represent mean levels ±SEM.

CLR specific antibodies were used to stain and profile DC subsets, MDDCs and mDCs, for expression of CLRs (Fig. 1). Phenotype and activation status of isolated mDCs was confirmed after each isolation (Supplementary Figure 1). Both CD205 (DEC-205) and CD206 (MMR), type I CLRs belonging to the mannose receptor (MR) family were expressed on MDDCs and mDCs. CD207 or langerin, type II CLR specific to Langerhans cells and CD303 or BDCA2, a human plasmacytoid DC marker were absent in both subsets. CD209 or DCSIGN (type II), a classic tissue-differentiated DC marker^25^ was predictably present on MDDCs but not mDCs (Fig. 1a). DCs also showed expression of ITIM (immunoreceptor tyrosine-based inhibitory motif) associated CLEC4A and CLEC12A receptors and ITAM (immunoreceptor tyrosine-based activation motif) associated CLEC9A receptor. Interestingly, mDCs showed elevated expression of CLEC12A. Low levels of CLEC10A were detected in both MDDCs and mDCs (Fig. 1b).

### C-type lectin receptors are important for binding and transmigration of DCs across brain microvascular endothelium in response to CCL2

In the multistep paradigm of leukocyte transmigration^21, 26^, while lectins can mediate the tethering and rolling of leukocytes to the vessel wall^27–29^, presence of a chemoattractant ensures the directional pull across the BBB thereby triggering firm attachment of DCs to the endothelial surface^26^. Others and we have previously shown expression of CCR2 on DCs and monocytes allows their CCL2-mediated transmigration^2^ and the ability to reactivate encephalitogenic T-cells during disease^30^.

**Figure 2:**
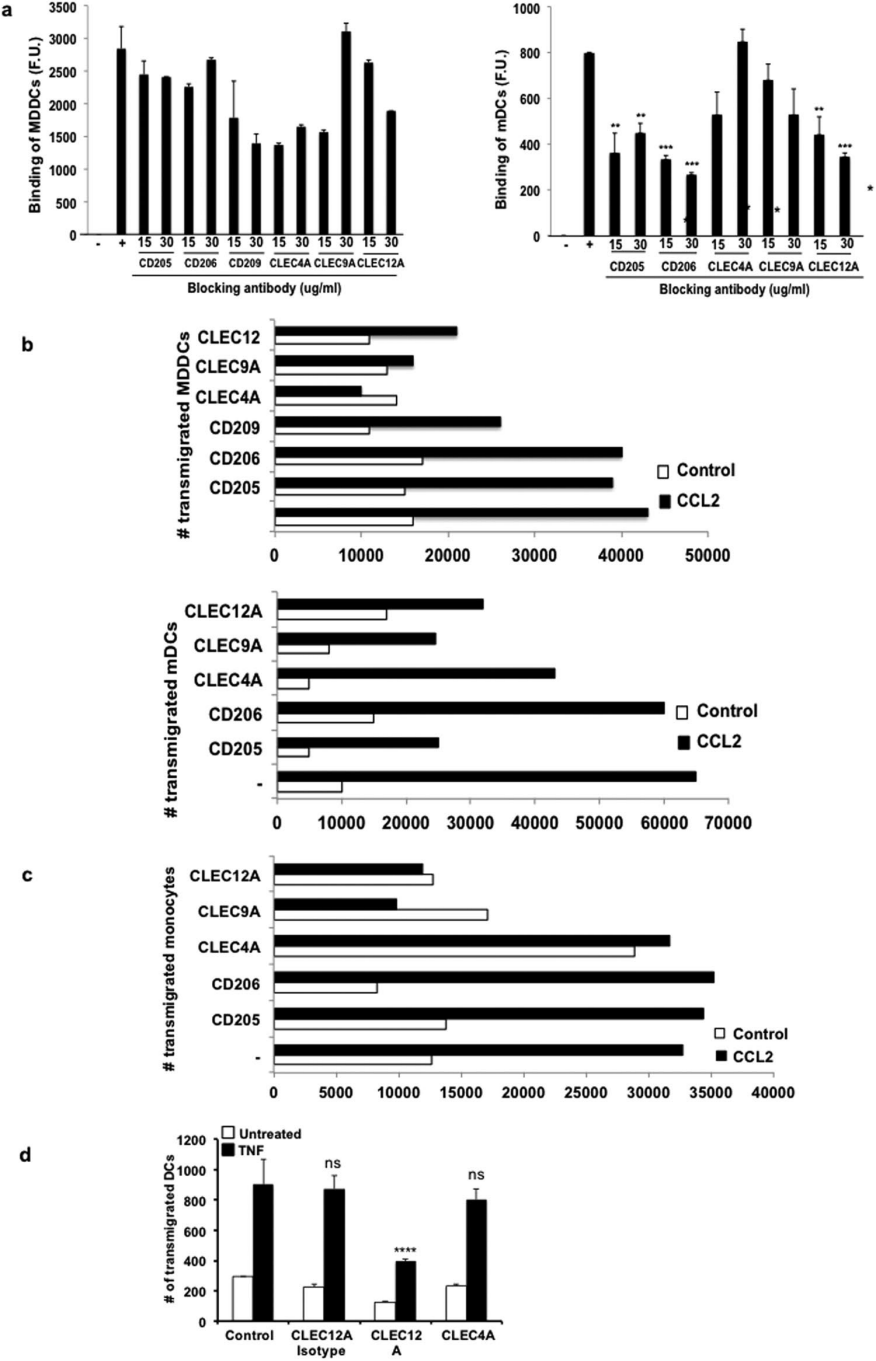
Effective CLR blocking impacts binding of DCs to brain endothelium and subsequent transmigration. DCs obtained from PBMCs were pre-treated with monoclonal antibodies fluorescently labeled prior to addition into a 96-well plate containing a confluent monolayer of TNF-α-activated hCMEC/D3 cells for 1 h and washed. Fluorescence was read at the 540 nm wavelength. For transmigration, pre-treated DCs and monocytes were added to the hCMEC/D3 cell monolayer and allowed to transmigrate for 24 h. Reduction in fluorescence intensities upon binding (**a**) and number of transmigrated cells (**b**) were observed in MDDCs and mDCs. (**c**) Bar graph showing number of migrated monocytes. (**d**) Similarly, migration of mouse splenic DCs across bEnd.3 cells is shown as a bar graph. Significance is represented when *P < 0.05, **P < 0.01 and ***P < 0.001. Bar graphs are representative of two independent experiments run in triplicate. (See also Figures S2 and S3).

Examination of MDDCs, both activated and non-activated, revealed more CCR2 expression in comparison with T cells (Supplementary Figure 2A). We then used TNF-α-activated hCMEC/D3 cells^31^- a brain microvascular endothelial cell line with many close characteristics of the primary cells^32^- and allowed fluorescent dye-labeled DCs to bind to them. hCMEC/D3 cells themselves do not show expression of CLRs of interest (Supplementary Figure 2B). Testing the blocking efficiency of antibodies showed that receptors became unavailable for binding (Supplementary Figure 2C). Blocking CD209 or DCSIGN, CLEC4A, CLEC9A and CLEC12A on DCs, all resulted in reduced fluorescence intensity, indicating decreased binding (Fig. 2a). For BBB set-up, MDDCs were added to activated hCMEC/D3 cells grown on membrane inserts in the presence of CCL2 and blocking antibodies. CCL2 did not have a direct effect on CLR expression on DCs (Supplementary Figure 2D). The BBB model demonstrated trans-endothelial electrical resistance (TEER) values in excess of 200 ohms/cm^2^_,_ suggesting the formation of a tight barrier. (Supplementary Figure 2E). For MDDCs, CD209, CLEC4A, CLEC9A and CLEC12A (Fig. 2b) receptors were important for transmigration. Similar experiments on mDCs, revealed that CD205 (p < 0.01), CD206 (p < 0.001) and CLEC12A (15ug, p < 0.01 and 30ug, p < 0.001) receptors are involved in attachment to the endothelium, whereas CD205, CLEC4A, CLEC9A and CLEC12A are important for transmigration. Further, monocytes also appeared to utilize CLEC9A and CLEC12A receptors in transmigration (Fig. 2c). CD4^+^ and CD8^+^ T-cells did not utilize these CLRs (Supplementary Figure 3A and B) to transmigrate and may solely rely on integrin adhesion^4, 33^). Further, upon using a murine system of the BBB model, we saw a similar reduction in DC migration across the endothelial layer (bEnd.3) upon CLEC12A blocking (Fig. 2d).

### SHP1/2 signaling is important for CCL2-driven migratory phenotype in DCs

**Figure 3:**
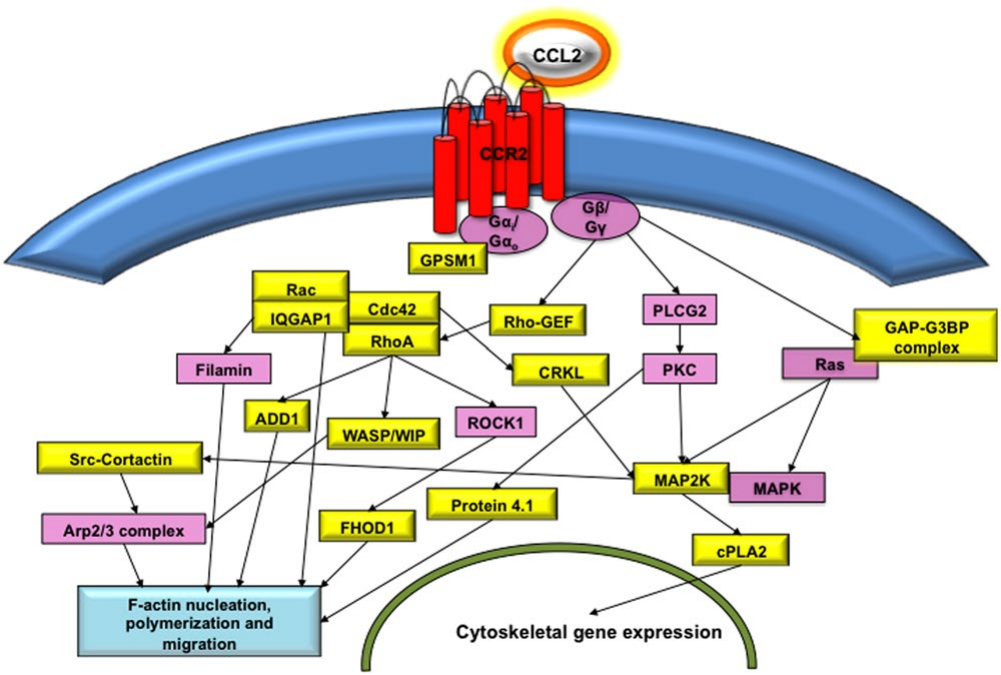
Phosphoproteomic analysis of CCL2 treated DCs and confirmation of relevant signaling pathways. Schematic of actin and MAPK related proteins from pathway analysis of identified proteins from the CCL2 treated samples (yellow- phosphorylated protein, pink- molecules linking identified phosphorylation events). (See also Figure S4).

**Figure 4:**
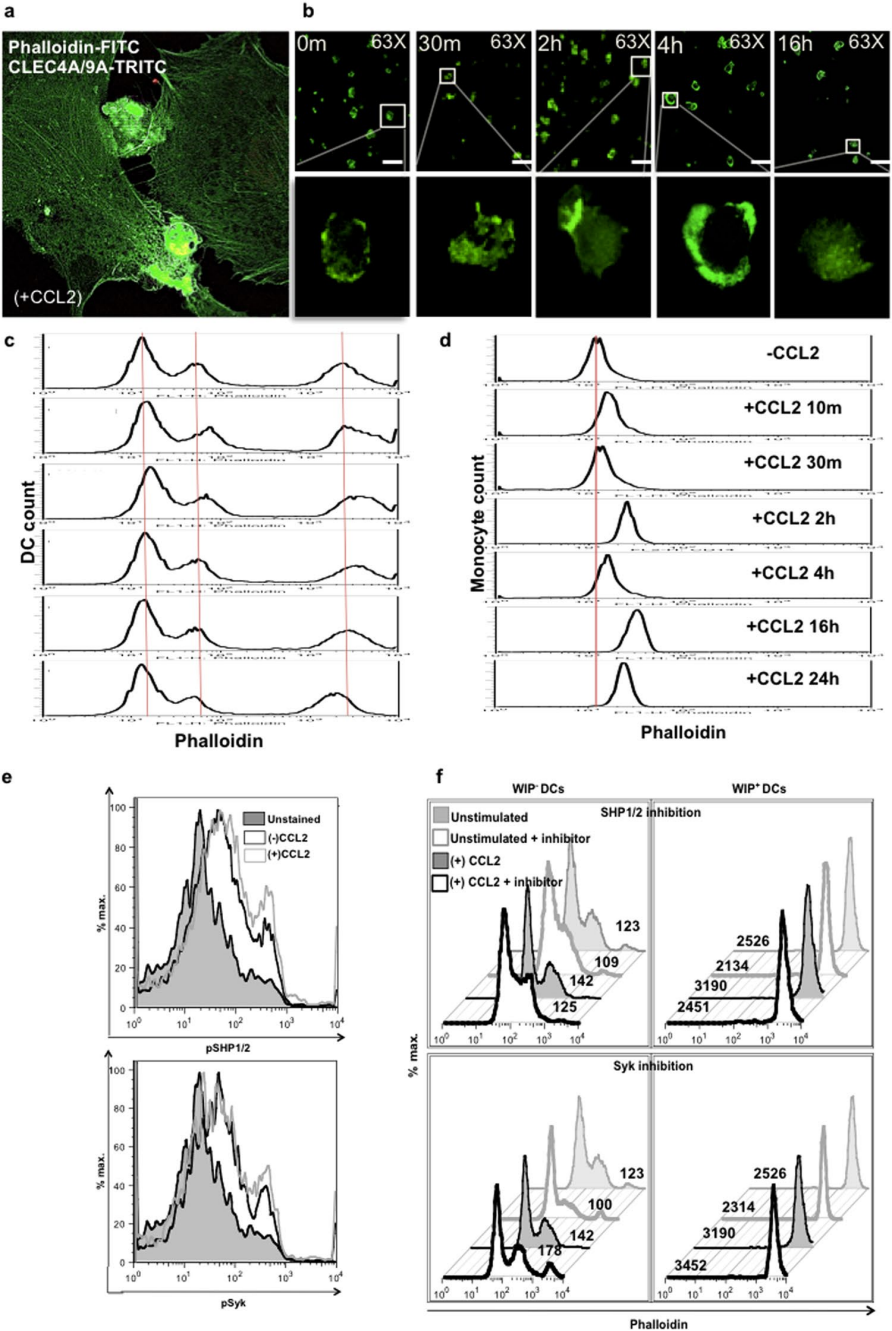
Increased actin polymerization dynamics in DCs upon CCL2 treatment and its decrease upon SHP1/2 inhibition. (**a**) PBMCs were added to a transwell containing a confluent layer of activated hCMEC/D3 cells in the insert and CCL2 (100 ng/ml) in the lower well. After 2 h, transwell was stained for phalloidin-FITC (3 ug) as well as TRITC labeled anti-CLEC4A, -CLEC9A antibodies. Microscopy evidence of intense F-actin expression on cells (co-labeled as yellow) as they appear to be transmigrating between two endothelial cells (green). Images are representative of several fields of vision taken on three separate transwells. (**b**) Confocal microscopy images indicate areas of actin polymerization on MDDCs treated with CCL2 (100 ng/ml) for indicated durations and labeled with phalloidin-FITC. All images were taken at 63X. Scale bar: 25 μm. Flow cytometry histograms show staining intensity of (**c**) MDDCs and (**d**) monocytes at different time points indicating more (right shift) or less (left shift) addition of actin subunits in reference to the control (red line). (**e**) Identification of SHP1/2 and Syk phosphorylation in MDDCs upon CCL2 treatment. (**f**) Phalloidin-FITC histograms on WIP^−^ and WIP^+^ MDDCs upon SHP1/2 inhibition (30 uM) for 3 h or Syk inhibitor (piceatannol, 30 uM) for 1 h. Histograms are representative of results from two individual donors. (See also Figure S5).

A concerted facilitation of CLR signaling within DCs and CCL2-driven chemoattraction is important for interactions with the BBB in order to enable neuroinvasion. In fact, analysis of the actin cytoskeletal molecular signaling pathway reveals MAPK and F-actin nucleation signaling molecules upon CCL2 treatment as summarized in Table 1 and Fig. 3 (derived from a phosphoproteomic analysis of various biological processes and molecular functions in Supplementary Figure 4A and 4B). CLEC4A^+^ and CLEC9A^+^ immune cells stained very brightly with phalloidin (a marker for F-actin nucleation), whereas the endothelial cell monolayer stained very diffusely (Fig. 4a) within a transwell system containing CCL2. Further, phalloidin expression on DCs (Fig. 4b) showed increased intensity within 30 m of CCL2 treatment. Besides DCs, only monocytes were (Fig. 4d) (Supplementary Figure 5) found to be responsive to CCL2 treatment.

**Table 1:** MAPK and F-actin nucleation signaling molecules upon CCL2 treatment.

Accession ID	Protein name	Peptide in treatment sample	Phosphorylation site	pSR	Xcorr	Pathway
P46734	Dual specificity mitogen-activated protein kinase kinase 3	mcDFGISGYLVDsVAK	M1(Oxidation); C2(Carbamidomethyl); S13(Phospho)	175	5.42	MAPK
P52564	Dual specificity mitogen-activated protein kinase kinase 6	McDFGISGYLVDsVAK	C2(Carbamidomethyl); S13(Phospho)	207	4.83	MAPK
O43353	Receptor-interacting serine/threonine-protein kinase 2	SPsLNLLQNK	S3(Phospho)	101	3.43	MAPK
Q13177	Serine/threonine-protein kinase PAK 2	DGFPSGTPALNAK	S5(Phospho)	96	3.56	MAPK
P47712	Cytosolic phospholipase A2	HIVSNDSSDSDDESHEPK	S10(Phospho)	89	4.22	MAPK
Q15418	Ribosomal protein S6 kinase alpha-1	KAYSFCGTVEYMAPEVVNR	C6(Carbamidomethyl); T8(Phospho)	62	4.68	MAPK
P51812	Ribosomal protein S6 kinase alpha-3	KAYSFCGTVEYMAPEVVNR	C6(Carbamidomethyl); T8(Phospho)	62	4.68	MAPK
P51812	Ribosomal protein S6 kinase alpha-3	NSIQFTDGYEVK	S2(Phospho)	89	3.93	MAPK
Q9UK32	Ribosomal protein S6 kinase alpha-6	KAYSFCGTVEYMAPEVVNR	C6(Carbamidomethyl); T8(Phospho)	62	4.68	MAPK
P46109	Crk-like protein	DSSTCPGDYVLSVSENSR	S2(Phospho); C5(Carbamidomethyl)	110	5.4	MAPK
P46940	Ras GTPase-activating-like protein IQGAP1	SKSVKEDSNLTLQEK	S3(Phospho)	53	4.8	actin
Q15052	Rho guanine nucleotide exchange factor 6	KDSIPQVLLPEEEKLIIEETR	S3(Phospho)	100	4.03	actin
Q14155	Rho guanine nucleotide exchange factor 7	MSGFIYQGK	S2(Phospho)	102	3.25	actin
P35579	Myosin-9	GAGDGSDEEVDGKADGAEAKPAE	S6(Phospho)	60	4.25	actin
Q13177	Serine/threonine-protein kinase PAK 2	DGFPSGTPALNAK	S5(Phospho)	96	3.56	actin
P62328	Thymosin beta-4	TEtQEKNPLPSK	T3(Phospho)	60	4.26	actin
A8MW06	Thymosin beta-4-like protein 3	TEtQEKNPLPSK	T3(Phospho)	60	4.26	actin
P46109	Crk-like protein	DSSTCPGDYVLSVSENSR	S2(Phospho); C5(Carbamidomethyl)	110	5.4	actin
P35611	Alpha-adducin	AAVVTsPPPTTAPHK	S6(Phospho)	100	3.32	actin
Q13884	Beta-1-syntrophin	GSPVSEIGWETPPPEsPR	S16(Phospho)	131	5.14	actin
Q13884	Beta-1-syntrophin	GsPQAGVDLSFATR	S2(Phospho)	140	3.25	actin
Q09666	Neuroblast differentiation-associated protein AHNAK	VSMPDVELNLKsPK	S12(Phospho)	90	3.94	actin
P13796	Plastin-2	FsLVGIGGQDLNEGNR	S2(Phospho)	127	3.93	actin
Q15149	Plectin	SSsVGsSSSYPISPAVSR	S3(Phospho); S6(Phospho)	113	5.01	actin
Q15149	Plectin	SsSVGsSSSYPISPAVSR	S2(Phospho); S6(Phospho)	102	5.01	actin
P11171	Protein 4.1	SPRPtSAPAITQGQVAEGGVLDASAK	T5(Phospho)	85	5.71	actin
P11171	Protein 4.1	SPRPTsAPAITQGQVAEGGVLDASAK	S6(Phospho)	93	5.71	actin
Q9NYL9	Tropomodulin-3	DLDEDELLGNLsETELK	S12(Phospho)	169	5.81	actin
P47736	Rap1 GTPase-activating protein 1	AAGISLIVPGKsPTR	S12(Phospho)	110	4.01	actin
Q14247	Src substrate cortactin	LPSsPVYEDAASFK	S4(Phospho)	114	3.24	actin

CLEC12A contains a single ITIM in its cytoplasmic tail that associates with the signaling phosphatases SHP1/2 leading to immune modulation. MDDCs, which showed increased phosphorylation of both SHP-2 and Syk upon exposure to CCL2 (Fig. 4e), were treated with inhibitors to both SHP1/2 and Syk and analyzed for phalloidin expression. Interestingly, inhibition of SHP1/2 reduced CCL2-induced actin polymerization, particularly on DCs expressing WASP-interacting protein (WIP) (Fig. 4f), a molecule known to associate with Wiskott–Aldrich syndrome protein (WASP) on DC podosomes^34^. Syk inhibition increased phalloidin expression (Fig. 4d, lower panel). This evidence suggests that ITIM regulated SHP1/2 phosphorylation is important for CCL2 driven migratory response.

### Administration of anti-CLEC12A antibody shows significant delay of disease onset, attenuation in clinical symptoms and relapse

**Figure 5:**
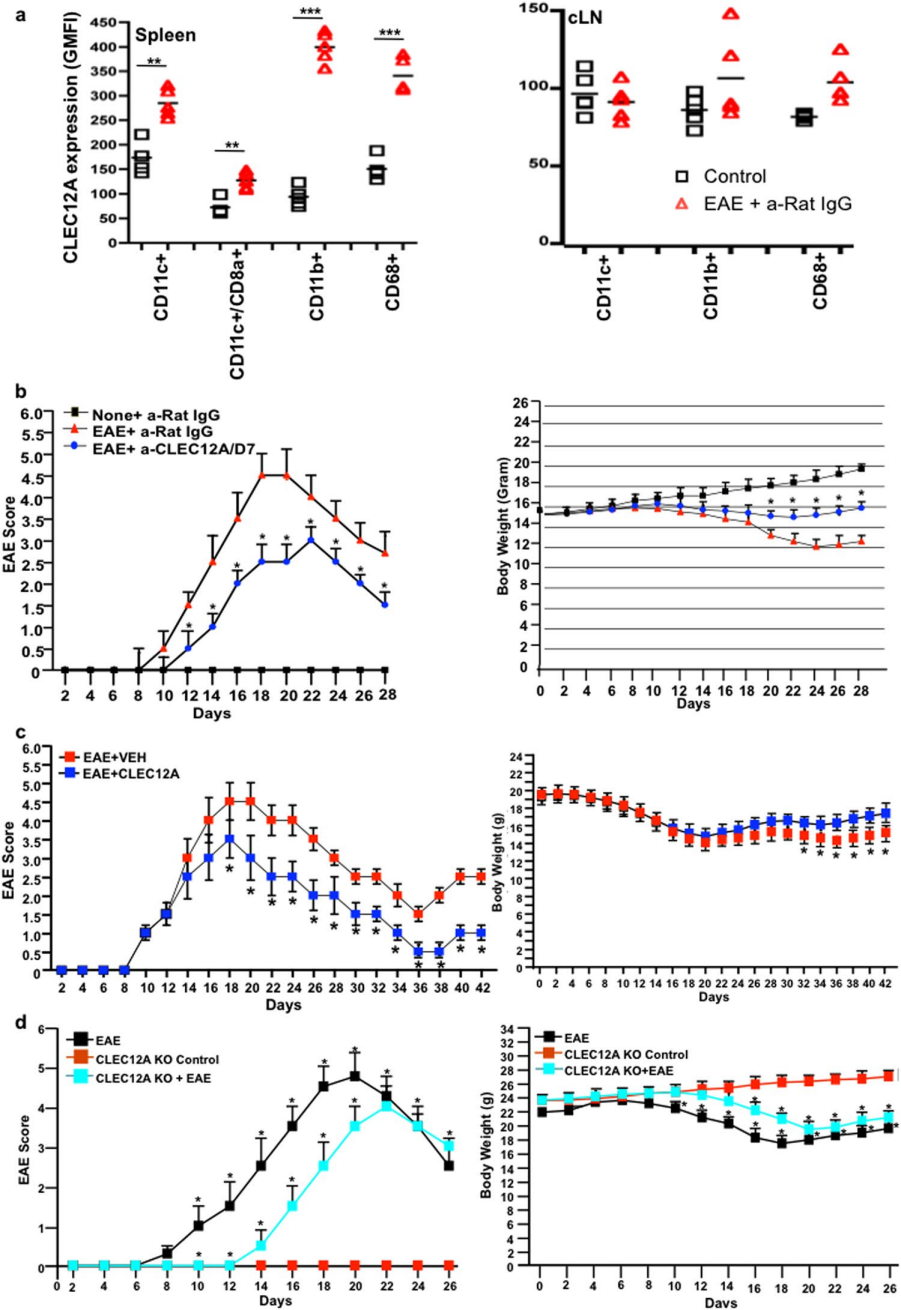
Impact of anti-CLEC12A antibody treatment on EAE severity and restoration in body weight. (**a**) Splenocytes and cLN cells from control and EAE mice were phenotyped for immune cell markers and further stained individually for CLEC12A expression. Plots represent CLEC12A GMFI levels of all animals in each group analyzed (n = 5) with the mean GMFI expression represented for each marker. (**b**) Data points indicating mean (n = 5) clinical disease score of C57BL/6 mice from control, EAE + IgG isotype and EAE + anti-CLEC12A antibody treatment on Day 7 (left panel). The body weight of control, EAE and Day 7 treated mice are shown on the right. (**c**) Data points indicating mean (n = 5) clinical disease score of SJL/J mice from EAE + vehicle and EAE + CLEC12A antibody treatment (Day 14 & Day 21) (left panel). The body weight of EAE and anti-CLEC12A antibody-treated mice are shown on the right. (**d**) Data points indicating mean (n = 5) clinical disease score (left) of C57BL/6 mice with EAE and C57BL/6 mice with/without CLEC12A and with/without EAE. The body weights of mice are shown on the right. *P < 0.01 and **P < 0.005.

Based on evidence from our *in vitro* studies, we wished to study the effects of blocking the ITIM bearing SHP1/2 associated CLR, CLEC12A, on myeloid immune cells, particularly DCs, on the clinical course of EAE in C57BL/6 mice after immunization with myelin peptides. Notably, EAE mice showed significantly higher levels of CLEC12A expression on myeloid cells within the spleen and cervical lymph nodes (cLNs) compared to control mice (Fig. 5a). Upon MOG_35–55_ peptide immunization, C57BL/6 mice showed disease onset on Day 9 that progressively peaked on Days 18–19. To block early invasion of DCs into the CNS, anti-CLEC12A antibody was administered on Day 7 prior to disease onset. First, antibody specificity was tested on *ex vivo* spleen sections from CLEC12A^−^/^−^ C57BL/6 mice wherein no staining was observed as opposed to the sections from wild-type mice (Supplementary Figure 6). Alternatively, CLEC4A staining was seen in both the KO and wild-type sections. Anti-CLEC12A antibody treatment not only delayed onset by 2 days, but also significantly attenuated disease severity. Additionally, 50% of body weight was regained after antibody administration (Fig. 5b, right panel).

Since most patients have relapse remitting-MS (RR-MS), we injected anti-CLEC12A antibody after disease onset in a mouse model of RR-EAE (Fig. 5c, left panel). Clinical behavioral scores for mice treated with anti-CLEC12A antibody on Day 14 and Day 21 suggest quite significantly alleviated severity of PLP_139–151_-mediated disease progression. Remarkably, Day 36 showed significant remission upon treatment compared to untreated mice. Thereafter, a significantly higher relapse score was seen in untreated versus treated mice. Figure 5c (right panel) demonstrates concomitant increase in body weight as treatment alleviates disease symptoms. Importantly, induction of EAE in CLEC12A^−^/^−^ mice revealed a 7 day delay in disease onset along with reduced disease severity (Fig. 5d).

### Anti-CLEC12A antibody treatment reduces DC infiltration within CNS of EAE mice

**Figure 6:**
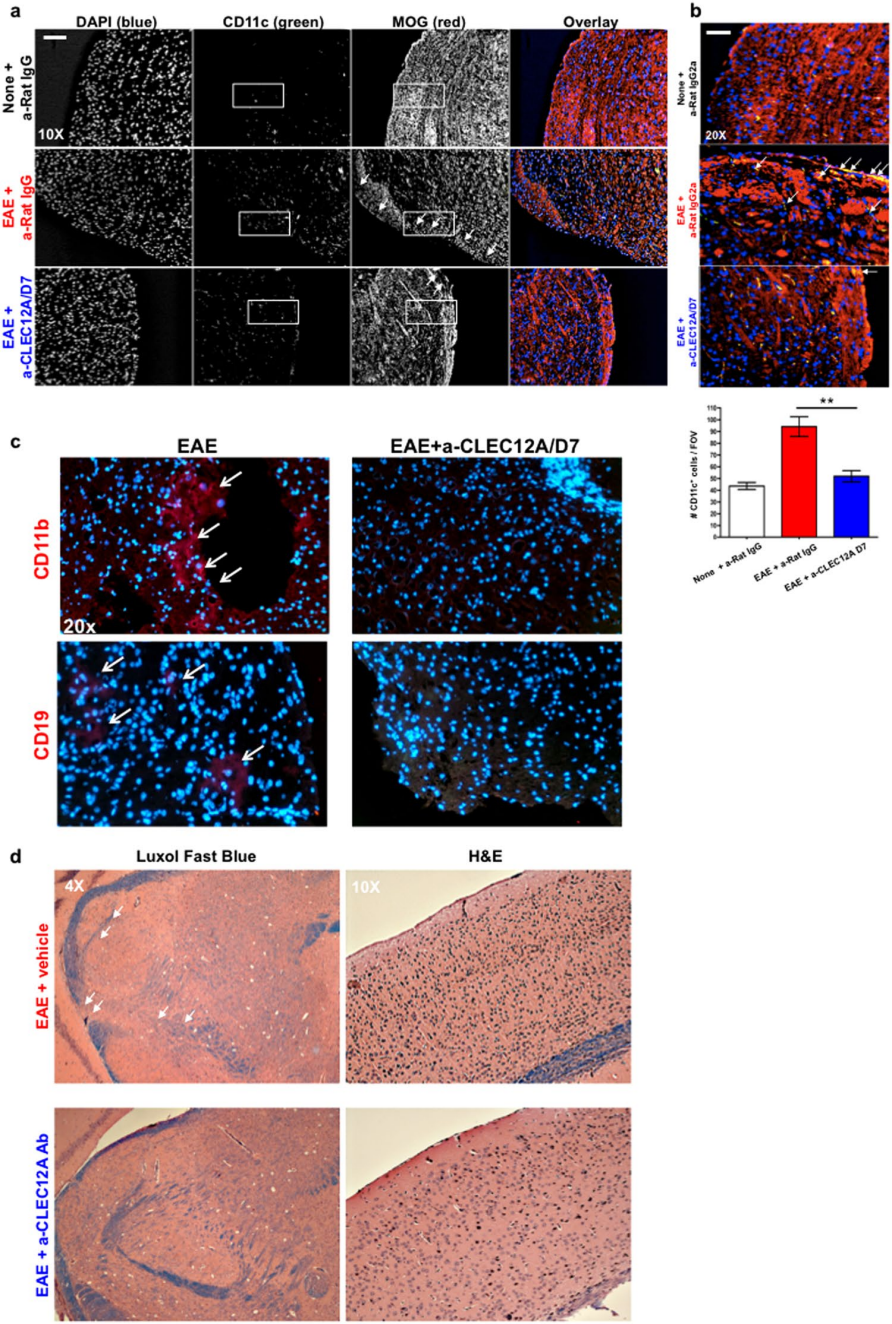
CLEC12A antibody treatment blocks DC infiltration within CNS tissue of EAE mice. (**a**) Spinal cord tissue from C57BL/6 mice was subjected to immunoflorescence staining with anti-CD11c (green), anti-MOG antibodies (red) and DAPI (blue). Images show demyelination (white arrows) and visual enumeration of CD11c^+^ DCs (white box) in areas of MOG staining at 10X resolution from control, EAE and Day 7 anti-CLEC12A treated mice. Numbers represent counts from ten fields of vision from three to four sections per mouse. (**b**) CD11c^+^ DC infiltration in areas near blood vessel of spinal cord. (**c**) Spinal cord tissue from EAE and Day 7 anti-CLEC12A treated mice C57BL/6 mice were subjected to immunoflorescence staining with anti-CD11b (Red), anti-CD19 (Green) antibodies and DAPI (blue) for myeloid cell infiltration. (**d**) LFB and H&E staining from SJL/J brain tissue depicting areas of myelinalion (blue) and cellular infiltration (black), respectively. Data presented is representative of two mice per group. For all 10x and 20x images, Scale bar: 100 μm and for 4x images, Scale bar: 200 μm

Histopathology on lumbar spinal cords taken from isotype-treated mice undergoing EAE displayed irregular myelin oligodendrocyte staining indicating severe demyelination as compared to control mice (Fig. 6a). CD11c^+^ positive DCs in the spinal cord accumulated in much higher numbers in areas with ongoing demyelination as also seen before^2^. Remarkably, Day 7 anti-CLEC12A antibody treatment shows preservation of myelination and significantly lesser accumulation of DCs. EAE tissues indicated dramatic DC infiltration and direct association with myelin (Fig. 6b), which was much less evident upon anti-CLEC12A antibody treatment. Additionally, infiltration of CD11b^+^ myeloid cells such as macrophages and CD19^+^ B cells was also markedly reduced in the Day 7 CLEC12A antibody treated animals (Fig. 6c). Brain tissue of SJL/J mice also revealed increased preservation of myelin and less cellular infiltration upon anti-CLEC12A antibody treatment compared to no treatment (Fig. 6d).

### CLEC12A antibody treatment retains and restores DC function in the periphery in EAE mice

**Figure 7:**
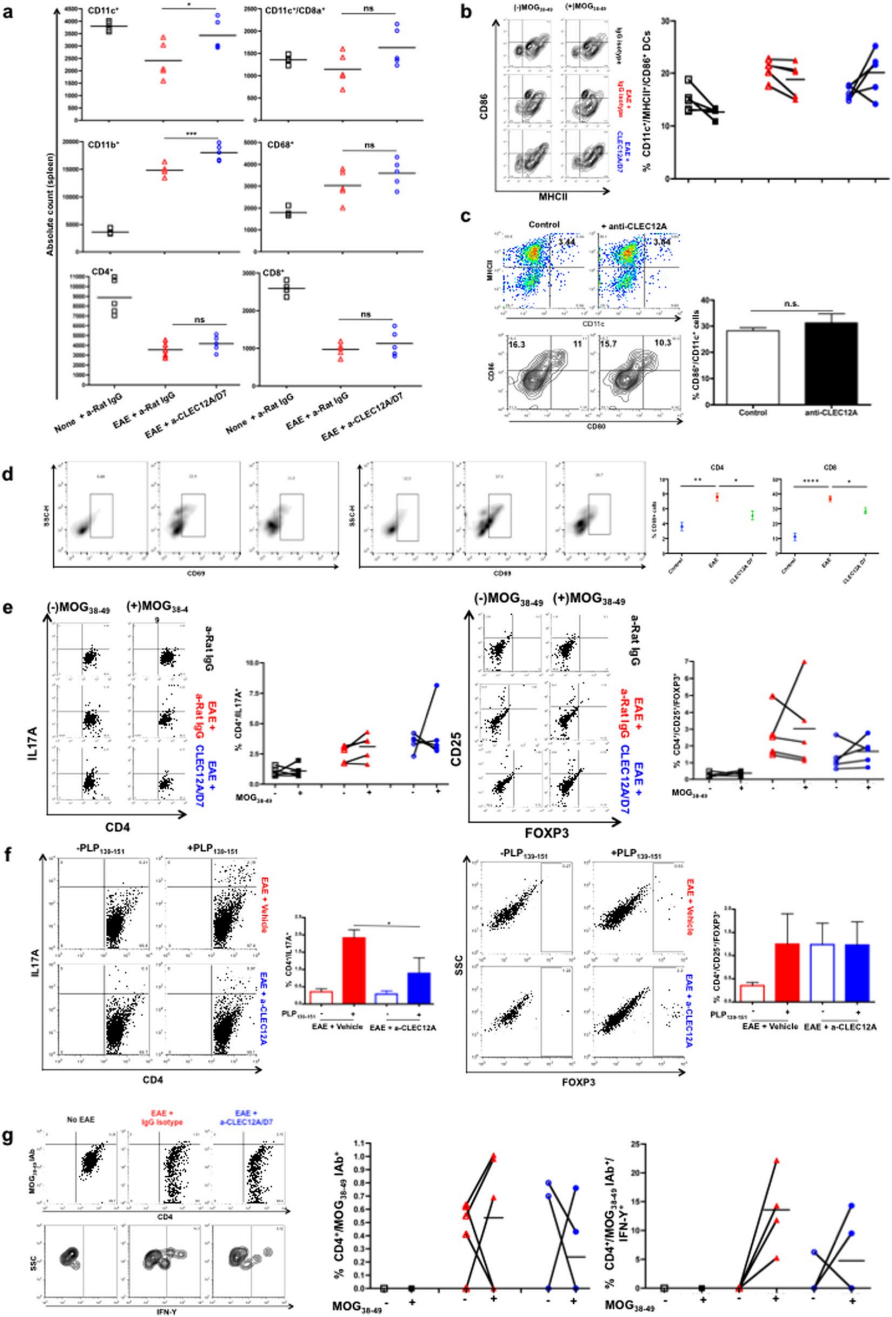
Quantification and functional analysis of myeloid cells within the spleen upon CLEC12A antibody treatment of both progressive and relapse-remitting EAE mice. (**a**) Splenocytes from C57BL/6 mice with control IgG isotype, EAE + IgG isotype and EAE + CLEC12A antibody treatment (Day 7) were stained for indicated immune cell markers for quantification. Each point represents absolute count of each individual marker for each animal in every group analyzed (n = 5) with a bar that represents mean count for each marker. (**b**) CD11c^+^ cells expressing both MHCII and CD86 from splenocytes with and without MOG_35–55_ stimulation for 3 days followed by activation with cell activation cocktail A for 5 h. Each point represents percentage of each individual marker for every group analyzed (n = 5) with a bar that represents mean percentage for each marker (right). Flow cytometric contour plots (left) showing one representation of MHCII^+^/CD86^+^ co-expression for all groups of mice. (**c**) Splenocytes from three mice were also evaluated for MHCII^+^ expression on CD11c^+^ cells and CD80 and CD86 markers upon no treatment and anti-CLEC12A antibody treatment. Representative expression is shown. (**d**) Splenocytes from five mice were evaluated for CD69^+^ expression on CD4^+^ and CD8^+^ T-cells upon no treatment and anti-CLEC12A antibody treatment. Representative expression is shown. Flow cytometry analysis representing CD4^+^ cells expressing IL17A (top) and CD25^+^/FOXP3^+^ (bottom) from C57BL/6 and SJL/J mice upon (**e**) MOG_35–55_ and (**f**) PLP_138–151_ stimulation respectively for 3 days followed by activation for 5 h. Each bar represents mean percentage for every marker per group (n = 5). Representative flow cytometry dot plots from one animal per group are shown on the left. (**g**) Flow cytometry analysis representing CD4^+^ cells expressing MOG_38–49_ IAB^+^/IFN-Υ^+^ from control anti-Rat IgG2a, EAE + anti-Rat IgG2a and EAE + CLEC12A antibody-treated (Day 7) C57BL/6 mice with and without MOG_35–55_ stimulation for 3 days followed by activation for 5 h. Each point represents percentage of each individual treatment for every group analyzed (n = 5) with a bar that represents mean percentage for each marker (bottom). *P < 0.01 and **P < 0.005.

Upon quantification of immune cells of C57BL/6 mice, splenocytes revealed higher numbers of CD11c^+^, CD11c^+^/CD8a^+^ and CD11b^+^ cells within anti-CLEC12A antibody-treated mice compared to isotype-treated EAE mice, suggesting accumulation in the periphery (Fig. 7a). A similar increase in the number of DCs was observed in the cLNs upon antibody treatment (Supplementary Figure 7A). This upregulation was not seen amongst myeloid cells found within splenocytes of SJL/J mice (data not shown). Interestingly, we found a higher percentage of CD11c^+^ DCs expressing CCR2 in the untreated mice (Supplementary Figure 7B) consistent with a study showing higher chemoattractant receptor expression in mDCs of MS patients^35^. Treatment with anti-CLEC12A antibody was seen to reduce CCR2 expression on these cells (Supplementary Figure 7B). Splenic CD4^+^ and CD8^+^ T-cells were not directly affected in numbers upon antibody treatment (Fig. 7a), however, there was a significant decrease in their numbers in the cLNs suggesting reduced T cell trafficking in the brain (Supplementary Figure 7A). Similarly, our analysis of SJL/J mice revealed that the absolute number of CD11c^+^ DCs increased within peripheral blood along with a reduction in their CCR2 expression levels (Supplementary Figure 7C,D, and E). CD86 and MHCII expression on DCs in blood and periphery of the treated mice did not change indicating that anti-CLEC12A antibody did not have an impact on activation of DCs (Fig. 7b). This was further corroborated with *in vitro* exposure of CD11c^+^ cells with anti-CLEC12A antibody (Fig. 7c) where neither CD86 or CD80 levels were affected by treatment.

Next, investigation into effect of the antibody on CD4 and CD8 T cell activation revealed that T cells within the spleen expressed higher CD69, which was reduced by antibody treatment (Fig. 7d). Th17 and T regulatory CD4 T cell phenotypes on splenocytes from anti-CLEC12A antibody-treated EAE mice exhibited reduced IL-17A^+^ cells compared to untreated EAE mice (Fig. 7e). A closer look at individual responses show that numbers of IL-17A^+^/CD4^+^ T-cells and CD25^+^/FOXP3^+^ CD4^+^ T-cells decreased moderately but not significantly between untreated and treated EAE mice upon MOG stimulation. This is perhaps due to the fact that splenocytes were harvested from mice where remission had already set in. SJL/J mice, however, indicated a significant decrease in splenic IL17A^+^ cells suggesting a dampened TH17 response upon restimulation with PLP_139–151_ peptide in treated mice, and constitutive high levels of regulatory T-cells as seen by increased FOXP3 expression (Fig. 7f). MOG_38–49_-specific CD4^+^ T-cells, however, showed a reduction in IFNγ (Fig. 7g).

## Discussion

CD11c^+^ DCs have been shown to be sufficient to reactivate myelin-specific T cells thus initiating the autoimmune demyelinating disorder characterized by the EAE model^36^. Further, antigen presentation by myeloid DCs has been implicated in driving progression of relapsing EAE^37^. CLEC12A, highly expressed on both mouse and human DCs, contains a single ITIM in its cytoplasmic tail that can associate with the signaling phosphatases SHP-1 and SHP-2 and seemed to be important for the migratory phenotype seen in DCs. Phosphoproteomic analysis of MDDCs upon CCL2 treatment showed that both MAPK and WIP/WASP protein complexes were phosphorylated. The actin cytoskeletal organization of podosomes is based on a WASP- and Arp2/3-mediated mechanism and it is the intact WIP–WASP complex that stabilizes DC podosomes^34, 38^. Upon inhibition of SHP-1/2 phosphorylation, there was decreased actin polymerization on WIP^+^ DCs. Mice administered with the anti-CLEC12A antibody both prior as well as after disease onset showed significant attenuation of disease with decrease in demyelination and CD11c^+^ DC infiltration. Analysis of peripheral organs in antibody administered mice showed a restoration of spleen DC levels, decrease in CCR2 expression, and a peptide specific response steering T cell proliferation away from the autoimmune TH17 response. In both strains of mice, we targeted CLE12A after the priming of T cells by APCs and when migration of T cells and APCs to the CNS has taken place^3^. CLEC12A, being a C-type lectin receptor can also play a role in uptake of self-antigen and presentation to T cells during disease initiation. In knockout mice, delayed disease induction indicates that this receptor may be important for antigen uptake as well as disease propagation. However, induction of EAE can also be initiated and propagated by other cell types within the CNS such as the microglia and we are able to see initiation of disease albeit delayed. Absence of CLEC12A in mice can give rise to other compensatory mechanisms, upregulating other C-type lectin receptors that can help in antigen uptake and binding of cells to the microvascular endothelium, allowing cells to migrate and maintain a diseased state *in vivo*. Therefore, targeting CLEC12A during ongoing disease is more effective in curbing inflammation due to its relevance during diseased state.

Here, we found CLEC12A to be involved in binding and transmigration of DCs across the BBB. CLEC12A is expressed on cells of myeloid lineage including monocytes, macrophages and DCs^39–42^, validating it as a myeloid-specific target for further study. Interestingly, CLEC12A is highly N-glycosylated and the degree of glycosylation varies significantly in different leukocyte populations^42, 43^, which may ultimately have functional consequences for ligand binding. Not long ago, CLEC12A was shown to sense monosodium urate microcrystals (MSU) from dying cells thereby responding to noninfectious inflammation, giving this receptor importance in autoimmunity and inflammatory disease^44^. However, bone marrow, spleen, and kidney all possess endogenous ligands to CLEC12A, that are yet to be characterized^43^. Our data suggests that there is a ligand on endothelial cells of the BBB as well.

A more recent study showed that CLEC12A^−^/^−^ mice develop exacerbated disease in a collagen antibody-induced arthritis (CAIA) model. Antibody administration against CLEC12A in wild type mice also resulted in a similar phenotype^45^. However, CAIA is highly driven by autoantibody effects unlike MOG-peptide induced EAE^46^. CLEC12A as a regulator of inflammatory control was first demonstrated when i.p. injections of MSU and subsequent injections with dead kidney cells induced inflammatory responses in the mice^44^. Here, hyper responsiveness from MSU injections is unlike EAE where high serum uric acid (UA) levels has been shown to reverse the disease progression^47, 48^ due to a protective role of UA in EAE and MS^49, 50^ owing to its peroxynitrite scavenging activity^51, 52^. Blocking of CLEC12A could thus prove beneficial in maintaining UA levels resulting in neuroprotection in addition to the vivo-produced benefits due to the impaired migration ability of DCs.

Phosphorylation of CLEC12A ITIM receptor allows recruitment of SH2 domain-containing protein tyrosine phosphatases (PTP), such as -1, SHP-2, and SHIP that dephosphorylate various protein tyrosine kinases, adaptor molecules, or enzymes to balance or suppress the activating signaling. However, it is now acknowledged that ITIM mediated SHP-1 and SHP-2 signaling, including signaling through CLEC12A, can have activating properties including phosphorylation of p38MAPK, ERK, increased cytokine expression, upregulation of CCR7, TLR2 and TLR4, thus indicating increased DC maturation, migration and antigen processing^44, 53–57^. A recent study has shown another such phosphatase, PTPN12 is important for the migration of DCs^58^. However, the exact events following antibody binding to CLEC12A are yet to be elucidated. Binding of antibody may lead to receptor neutralization thereby making it unavailable to bind to ligands at the BBB and inhibit migration and activation pathways. It may also lead to receptor internalization and subsequent activation of the CLEC12A receptor resulting in phosphorylation of SHP thereby triggering a slew of anti-inflammatory molecules and an inhibition of function (Supplementary Figure 8).

Our study brings forward a clinically viable target to inhibit myeloid cell migration. It is now acknowledged that CNS-infiltrating DCs are crucial for restimulation of co-infiltrating T-cells^2, 55, 58, 59^. So far, specific depletion of β1 integrins on DCs has shown reduced adhesion of DCs to the BBB^20^, but non-selectively. Hence, we believe efforts should be now spent in devising therapies based on myeloid-specific cell contacts, namely the CLRs investigated here, as these molecules have been historically studied for their roles in adhesion to cells and pathogens^22^ and can serve as promising candidates to curb the propagation of inflammation within the CNS.

## Methods

### Isolation of dendritic cells from blood

Peripheral blood mononuclear cells (PBMCs) were isolated from heparinized blood (Biology Speciality Corporation) by Ficoll-Paque Plus (Amersham Biosciences) density gradient centrifugation. Monocytes obtained by the adherence method from PBMCs were cultured in 1% normal human plasma (Sigma-Aldrich) in the presence of rhGM-CSF (100 IU/ml; PeproTech) and rhIL-4 (300 IU/ml; PeproTech) for 5 days. Cells were provided with fresh cytokines every other day. pDCs and mDCs isolation from PBMCs was carried out by magnetic separation with a MACS Separator (Miltenyi Biotec) according to manufacturer’s protocol.

### Flow cytometric phenotyping of CLRs on MDDCs and isolated mDCs

The purity of MDDCs and mDCs were verified by flow cytometry, and was shown to be ~90% pure for CD11c and CD1c respectively. The DCs were stained for C-type lectins using anti-CD205, -CD206, -CD207, -CD209, -CLEC4A, -CLEC9A, -CLEC10A, -CLEC12A (Biolegend) and -CD303 (Miltenyi Biotec) antibodies conjugated to PE. FACS data was acquired on BD FACS Calibur (BD Biosciences, San Jose, CA, USA) and analyzed with FlowJo software (Tree Star).

### Dendritic cell-brain endothelial cell adhesion assay

Human endothelial cells (hCMEC/D3) obtained from Dr Pierre-Olivier Courard (Institut Cochin, Paris, France) were seeded into collagen-coated (50 μg/ml; Trevigen) wells of a 96-well microplate (BD Biosciences) in complete EBM-2 media (Lonza) until 100% confluency was reached. TNF-α (R&D Systems) (100 U/mL) was also later added to some endothelial cell layers for 8 h to simulate the inflamed BBB through upregulation of receptor molecules.

MDDCs and mDCs were treated with varying doses (15, 25, 50 μg/ml) of specific anti-lectin antibodies namely, anti- CD205, CLEC4A, CLEC9A (R&D systems) and CD206, CLEC12A (Biolegend), CD209 (BD Biosciences) and incubated for 1 h for blocking these receptors. Untreated cells were set aside to be used as positive controls. DCs were then labeled with calcein AM fluorescent dye (5 μl/ml; Invitrogen) and added to each well of endothelial cells (non activated and activated) and incubated for 1 h to allow for binding to take place. A separate well was filled with 300,000 DCs, acting as a positive control for the fluorescent value of the total cells originally added. After 4 washes with RPMI, wells were filled with PBS and fluorescence was read by a multi-well plate reader (BioTek) at an excitation of 494 nm and an emission of 517 nm. Values were obtained and plotted as fluorescence unit (F.U.) from triplicate data, which was then statistically analyzed using a student’s t test to compare the difference in binding levels between the control and the experimental groups.

### Transendothelial migration assay

1 million primary MDDCs, mDCs and PBMCs cells were transferred to the upper chamber of polyethylene tetraphthalte transwells in the monolayer BBB model and allowed to transmigrate for 24 h across TNF-α (100 U/ml) activated endothelial cell layers grown on 8-micron membrane inserts. Cells were first treated with varying doses (15 and 30 μg/ml) of lectin blocking antibody as indicated previously and incubated for 1 h. Where indicated, CCL2 (R&D Systems) was added to the lower chamber (100 ng/ml) at the same time as immune cells were added to the upper chamber. At 24 h, transmigrated cells from the bottom chamber were removed and counted by trypan blue exclusion. Similarly, murine splenic DCs were isolated using the EasySep^TM^ Mouse CD11c Positive Selection Kit (STEMCELL Technologies) and 1 million Calcein AM labeled DCs were added to the upper chamber of the transwells in the presence of blocking antibodies against CLEC12A, CLEC12A isotype control and CLEC4A (30 μg/ml; R&D Systems) and allowed to transmigrate for 2 h across murine endothelial cells, bEnd.3 (ATCC), that were grown and activated on 8-micron membrane inserts. At 2 h, transmigrated cells were imaged using an inverted fluorescent microscope and the images were analyzed using ImageJ.

### CCL2-driven SHP1/2 and Syk phosphorylation and actin polymerization

MDDCs were treated with CCL2 for 30 m, collected, fixed, permeabilized and stained with anti-SHP-2 (pY542) and anti-Syk (pY348) antibodies (BD Biosciences) to detect phosphorylation. For actin polymerization detection, CCL2 treated MDDCs were also stained with phalloidin-FITC (Sigma-Aldrich) for 40 m at RT in the dark. Cells were also given Syk inhibitor (piceatannol, 30 uM, InvivoGen, San Diego, CA) for 1 h or SHP1/2 inhibitor (30 uM, EMD Millipore) for 3 h, then CCL2 (100 ng/ml) for 30 m. Cells were then stained with anti-WIP (pS488) antibody (BD Biosciences) and phalloidin to check phalloidin levels of podsomal DCs. Phallodin levels were specifically analyzed on WIP^+^- and WIP^−^-gated CD11c^+^ MDDCs.

### Sample preparation and phosphopeptide enrichment

Cell lysates were prepared from untreated and CCL2 treated (30 m) MDDCs. The cells were lysed in cell lysis buffer (Thermo Scientific) in the presence of protease inhibitor (Complete; Roche) and phosphatase inhibitor (PhosphoSTOP; Roche). After brief sonication and centrifugation, the soluble protein supernatants were separated from the insoluble debris. Protein concentrations were measured by Bradford protein assay (Bio-Rad). 400 μg of protein from control and treated was digested using the modified FASP procedure (filter aided sample preparation). Lysates were placed on a 5Kda filter device and washed with 100 mM NH_4_HCO_3_ to remove detergent. Samples were reduced with 100 mM DTT and alkylated with 50 mM iodoacetamide. Denatured proteins were digested with sequencing grade trypsin (Promega) in solution using a protein to trypsin ratio of 50:1 at 37 °C chamber overnight. For phosphopeptide enrichment, the samples were desalted and enriched using TiO2 enrichment kit according to manufacturer’s instruction (Thermo Scientific). Each of the enriched peptide samples was desalted and stored at −20 °C before analysis.

### Phosphopeptide analysis by LC-MS/MS

Mass spectrometry experiments were carried out using LTQ-Orbitrap Velos instruments (Thermo Electron) interfaced with nano ultimate high-performance liquid chromatography (HPLC; Dionex). As a part of the online sample clean-up step, the peptides were first concentrated using a 300 μm ID × 5 mm C18 RP trap column (Dionex) and then separated using a 75 μm ID × 15 cm C18 RP analytical column (Dionex), equilibrated in 4% ACN/0.1% FA at 250nL/minute flow rate. Mobile phase A was 2% ACN and 0.1% FA in water, whereas mobile phase B was 0.1% FA and 90% ACN in water. Peptides were separated with a gradient of 4–50% B in 60 minutes and 50–80% in 90 minutes and eluted directly into the mass spectrometer. The mass range in MS mode was 350 Da–1500 Da and in MS/MS mode it was set as 100 Da–1500 Da. The peptides were analyzed using a data-dependent method. Examples of the mass spectometry data from phosphoproteomics of two such identified peptides (Supplementary Figure 5, MAP2K6-top panel and SRC8 cortactin-bottom panel) are shown. A total of 92 phosphopeptides were present in treatment sample as compared to 101 phosphopeptides present in control sample (Supplementary Tables 1 and 2). Among those proteins, 99 phosphopeptides with unique phosphorylation site from were identified in the treatment sample (Supplementary Table 3).

### Database search and pathway analysis

The acquired spectra data were searched against Swissprot protein database using Proteome Discoverer 1.3 (Thermo Scientific) to interpret data and derive peptide sequences with the following parameters: partial-trypsin specificity, peptide mass tolerance of ±20ppm, fragment ion mass tolerance of ±0.8 Da, maximum missed cleavages of 2, variable modifications of methionine oxidation, phosphorylation of serine, threonine, tyrosine and cysterine carbamidomethylation. The search results were filtered with cross-correlation (Xcorr) via charge states (+1:1.5, +2:2.0, +3:2.25, +4:3.0), delta correlation (ΔCn > 0.1). A stringent 1% false discovery rate threshold was used to filter candidate peptide, protein and phosphosite identifications. Individual phosphopeptide fraction datasets (common protein with common phosphorylation site in both groups or unique protein/common protein with unique phosphorylation site in treatment group) were collated and analyzed using Pathway studio software (Elsevier) to uncover biological processes and molecular functions that are over or under expressed amongst the cognate proteins corresponding to the CCL2 treatment (P < 0.01).

### Mice

Female C57BL/6 mice and SJL/J (6–8 weeks old) were purchased from the National Cancer Institute (Rockville, MD, USA). CLEC12A^−^/^−^ mice were generated as described previously^44^ and these mice and the corresponding wild-type controls were kindly provided by Drs. Konstantin Neumann and Jürgen Ruland from Institut für Klinische Chemie und Pathobiochemie, Technische Universität München, Munich, Germany.

### Ethics statement

Animals were housed at the AAALAC-accredited University of South Carolina, School of Medicine (Columbia, SC, USA). All animal procedures were performed according to NIH guidelines under protocols approved by the Institutional Animal Care and Use Committee of the University of South Carolina. All studies involving animals are in accordance with the ARRIVE guidelines for reporting experiments involving animals.

### Evaluation of anti-CLEC12A antibody in EAE immunized mice

Progressive EAE was induced in female C57BL/6 mice (6–8 weeks old) and the CLEC12A^−^/^−^ mice as described previously^60–62^. Briefly, 100 μL of 150 μg myelin oligodendrocyte glycoprotein (MOG_35–55_) (PolyPeptide Laboratories) peptide emulsified in complete Freund’s adjuvant (Difco) containing 4 mg/ml killed *Mycobacterium tuberculosis* (strain H37Ra; Difco) was injected subcuataneously. Following immunization, 200 ng of pertussis toxin (List Labs) was injected i.p. into mice on Day 0, followed by a 400 ng pertussis toxin i.p. injection on Day 2. Animals were randomized into groups and anti-CLEC12A antibody (100 μg; R&D systems) was administered i.p. on Day 7 after induction of EAE in one group of mice (n = 5). The anti-CLEC12A antibody used in this study was raised against Thr67-Arg267 peptide of mouse-myeloma cell line derived recombinant CLEC12A and has been previously used for the successful detection of murine CLEC12A^44^. IgG isotype (100 μg; R&D Systems) antibody was administered in naïve mice (n = 5) and EAE induced mice (n = 5) as a control. On Day 28, mice were sacrificed and spleen, cLNs, brain and spinal cord were harvested. For the KO studies, wild type and CLEC12A^−/−^ mice were immunized as described above, observed and scored for EAE symptoms and sacrificed on day 26. Paraffin-embedded sections (10 μm) were prepared from the spleen of the CLEC12A^**−/−**^ and wild-type mice for testing anti-CLEC12A antibody specificity. Immunofluorescence detection of CLEC12A, CLEC4A (320511; R&D Systems), and CD11c was carried out using antibodies against these markers.

Relapse-remitting EAE was induced in female SJL/J (6–8 weeks old). PLP_139–151_ peptide (50 μg) emulsified in complete Freund’s adjuvant (BD Diagnostics), containing killed *Mycobacterium tuberculosis* (400 μg/ml) was injected subcutaneously (50 μl per area). Animals were randomized into groups of EAE and anti-CLEC12A antibody treatment, which was administered i.p. on Day 14 and Day 21 after injection of PLP_139–151_. Mice were observed for EAE symptoms as well as remission/relapse. On day 42, i.e. shortly after first relapse, mice were sacrificed; spleen, cLNs, brain and spinal cord were harvested. Clinical scores (0, no symptoms; 1, limp tail; 2, partial paralysis of hind limbs; 3, complete paralysis of hind limbs or partial hind and front limb paralysis; 4, tetraparalysis; 5, moribund; 6, death) were recorded every 2 days for all experiments. The mean score was then calculated for each group.

### Histological analysis of demyelination and myeloid cell infiltration

CNS tissue from naïve + IgG isotype antibody, EAE + IgG isotype antibody or EAE + anti-CLEC12A antibody Day 7 were collected and fixed with 10% formalin and paraffin blocks were prepared. Microtome sections (10 μm) were generated and stained with 1:50 anti-CD11c (clone AP-MAB0806; Novus Biologicals), 1:100 anti-CD11b (polyclonal; Novus Biologicals), 1:50 anti-CD19 (6D5; Biolegend) and 1:50 anti-MOG antibody (polyclonal; Thermo Scientific). Brain tissue was further subjected to Luxol Fast Blue/Cresyl Violet staining (Novaultra), followed by hematoxylin & eosin (Polyscientific).

### Quantification of immune cells and stimulation with antigenic peptides

Spleen and cLNs were homogenized separately into a single-cell suspension and spleen tissue was subjected to red blood cell lysis. Cells were stained with anti-CD11c, anti-CD11b, anti-CD68, anti-CD4, anti-CD8a (Biolegend) and anti-CCR2 (R&D systems) antibodies for quantification and the presence of CCR2 receptor on each cell-type. Further, splenocytes and lymphocytes from control and EAE mice were phenotyped for CD11c, CD11c/CD8a, CD11b and CD68 immune cell markers and further stained individually for CLEC12A expression.

Splenocytes from mice with progressive EAE and RR-EAE were further cultured in a 24-well plate in the presence of 30 μg/mL MOG_35–55_ and PLP_139–151_ respectively for 3 days. Prior to harvest, splenocytes were stimulated with cell activation cocktail (Biolegend) containing phorbol myristate acetage (PMA), ionomyocin and brefeldin A. Cells were harvested and processed for anti-CD4, anti-IL-17A, anti-CD25 and anti-FOXP3 antibodies (Biolegend) staining using BD Cytofix/CytoPerm Fixation/Permeabilization Solution Kit. Cells were then acquired and analyzed for intracellular cytokines using the flow cytometer.

### Statistics

For comparisons between groups (control, EAE + IgG2a isotype antibody and EAE + anti-CLEC12A antibody), a two-tailed, unpaired nonparametric t test was used (Mann–Whitney). To determine the significance within a group, a two-tailed, paired nonparametric t test was used (Wilcoxon matched pairs test). The comparison was determined to be significant (*) if the P value was less than or equal to (≤) 0.05, very significant (**) if ≤0.01, and extremely significant (***) if ≤0.001. Statistical values were obtained using Prism version 4 C software (Graphpad Software Inc.).

## Electronic supplementary material

Supplementary Material
