# Supplementary material for: Antibody blockade of CLEC12A delays EAE onset and attenuates disease severity by impairing myeloid cell CNS infiltration and restoring positive immunity
Source: Sci Rep. 2017 Jun 2;7:2707. doi: 10.1038/s41598-017-03027-x (PMC5457463; doi:10.1038/s41598-017-03027-x)
Supplement: Supplementary file 1 — Supplementary Material [file 41598_2017_3027_MOESM1_ESM.pdf]

Antibody blockade of CLEC12A delays EAE onset and attenuates disease severity by impairing myeloid cell CNS infiltration and restoring positive immunity

Divya Sagar[1], Narendra P. Singh[2], Rashida Ginwala[1], Xiaofang Huang[4], Ramila Philip[4], Mitzi Nagarkatti[2,3], Prakash Nagarkatti[2], Konstantin Neumann[5], Jürgen Ruland[5], Allison M. Andrews[6], Servio H. Ramirez[6], Zafar K. Khan[1], and Pooja Jain[1]*

[1]Department of Microbiology and Immunology, Drexel University College of Medicine, Philadelphia, PA, USA
[2]Department of Pathology, Microbiology and Immunology, School of Medicine, University of South Carolina, Columbia, SC, USA
[3]William Jennings Bryan Dorn VA Medical Center, Columbia, SC, USA
[4]Immunotope Inc., Pennsylvania Biotechnology Center, Doylestown, PA, USA
[5]Institut für Klinische Chemie und Pathobiochemie, Klinikum rechts der Isar, Technische Universität München, Munich, Germany
[6] Department of Pathology and Laboratory Medicine, Lewis Katz School of Medicine, Temple University, Philadelphia, PA, USA

*Corresponding Author: Department of Microbiology & Immunology, and the Institute for Molecular Medicine & Infectious Disease, Drexel University College of Medicine, 2900 Queen Lane, Philadelphia, PA 19129, USA.
pjain@drexelmed.edu; 215-991-8393, Fax No.: 215-848-2271

Supplementary Figure 1. Analysis of purity and activation following mDC isolation. mDCs were isolated from human PBMCs using the MACS isolation kit and positively selected for CD1c expression. (A) Cells were stained with markers for B cells (CD19) and mDCs (CD1c, CD11c) before and after isolation to ascertain purity of CD19- $CD1c^+$ mDCs after isolation. (B) Lin1- cells were further stained with activation markers HLA-DR and CD86.

Supplementary Figure 2. Related to Figure 3. Expression of CCR2 and CLRs and the efficiency of the BBB model and blocking antibodies. (A) Highly purified monocyte-derived dendritic cells (MDDCs) were obtained from PBMCs of healthy donors by the adherence method. The adherent monocytes were cultured in 1% normal human plasma in the presence of rhGM-CSF and rhIL-4 for 5 days. MDDCs and PBLs were further activated for 24 hours with lipopolysaccharide and phytohemagglutinin, respectively. Non-activated and activated MDDCs and PBLs were analyzed for the surface expression of chemokine ligand 2 receptor (CCR2) by flow cytometry. Phenotyping analyses are representative of cells obtained from at least three donors. (B) Representative histograms of hCMEC/D3 brain microvascular endothelial cells showing individual C type lectin expression profile with expression levels (black) gated on isotype controls (gray). (C) MDDCs (top) and mDCs (bottom) were tested for blocking efficiency prior to addition to wells containing the endothelial cell monolayer to show that receptors were unavailable for binding to endothelium. (D) Myeloid DCs were phenotyped for CLR expression at 2h and 24h treatment with CCL2. Bar graphs depict comparative geometric mean fluorescence intensities (GMFI) of each CLR. (E) BBB model was set up as described in the Materials and Methods. 72 h after the establishment of the BBB, the integrity of the BBB formed on the insert membrane was also assessed by the measurement (Table) of trans-endothelial electric resistance (TEER). Blank cell culture inserts with no cells serve as control. The permeability of 1-cell (monolayer) systems was tested by treating cells with ethanol, growth medium and basal medium overnight. Inserts were then transferred to another plate and fresh medium were added to inserts. 150 uL of FITC-Dextran (1 mg/mL) was added to each insert and incubated for 12 hours. Medium from bottom chamber were collected and 100 uL were used to measure florescence using fluorescence plate reader with extinction at 480 nm and emission at 530 nm.

Supplementary Figure 3. Related to Figure 2. Generation of mass spectrometry peaks from phosphopreoteomics study. Cell lysates were prepared from CCL2 treated and untreated (30min) MDDCs. Phosphopeptide enrichment was performed as explained in Materials and Methods on 400ug protein from each condition mass spectrometry was carried out on these samples. (A) Gene ontology analysis was carried out post phosphopeptide enrichment and mass spectrometry analysis of protein lysate from CCL2 treated MDDCs to analyze functional (left) and biological (right) process involvement of identified proteins. Number following the pathway was the protein hits along the pathway. (B) Examples of mass spectrometry peaks generated for proteins a) MAP2K6 and b) SRC8-coractin depicting sequences with specific phosphorylation site.

Supplementary Figure 4. Effect of blocking CLRs on monocyte and T cell transmigration. hCMEC/D3 cells were grown and activated on 8-micron membrane inserts. PBMCs pre-treated with CLR blocking antibodies were added to the monolayer and allowed to transmigrate for 24h in response to CCL2 in the bottom chamber of the transwell. Transmigrated cells were stained with anti-CD14, -CD4 and -CD8 antibodies to calculate the number of transmigrating a) monocytes, b) CD8 and c) CD4 T-cells respectively, out of 30,000 acquired events.

Supplementary Figure 5. Related to Figure 4. Actin polymerization detection on T cells. Peripheral blood leukocytes (PBLs) were treated with CCL2 (100ng/ml) for different durations of 0m, 30m, 2hr, 4hr, 16hr and 24hr, fixed and labeled with phalloidin-FITC. Flow cytometry histograms (left panel) show degree of staining intensity of a) CD4 and b) CD8 T-cells at different time points indicating more (right shift) or less (left shift) addition of actin subunits in reference to the control (red line).

Supplementary Figure 6. anti-CLEC12A antibody specifically targets the CLEC12A protein on myeloid cell surface. CLEC12A KO mice were generated and splenic tissue was isolated from both the KO and WT mice followed by fixation and sectioning. The spleen sections were stained with anti-CLEC12A, anti-CLEC4A, CD11b and CD11c antibodies followed by fluorescent imaging. Sections show $CLEC12A^+$ (A), $CLEC4A^+$ (B) cells (yellow arrows) and myeloid cells (white arrows). For all 10x and 20x images, Scale bar: 100µm and for 40x images, Scale bar: 50µm

Supplementary Figure 7. Reduced CCR2 expression on DCs and their resultant accumulation in peripheral blood of antibody-treated EAE mice. (A) Cells of the cLNs from C57BL/6 mice with control anti-Rat IgG2a,

EAE+anti-Rat IgG2a and EAE+CLEC12A antibody treatment (Day 7 and 16) were stained for CD11c, CD11b, CD4 and CD8 markers for quantification. Each point represents absolute count of each individual marker for each animal in every group analyzed (n=5) with a bar that represents mean count for each marker. (B) Splenocytes from C57BL/6 mice with control anti-Rat IgG2a, EAE+anti-Rat IgG2a and EAE+CLEC12A antibody treatment (Day 7) were stained for CD11c, CD11b and CCR2 markers to plot CCR2 expression on DCs and monocytes. Bar graph represents percentage of CCR2 expression ($CCR2^{high}$ and $CCR2^{low}$) for group analyzed (n=5) with standard error bars. (C) Cells from peripheral blood of SJL/J mice with and without CLEC12A antibody treatment were stained for CD11c, CD11c/CD8a, CD11b, CD68, CD4 and CD8 immune cell markers for quantification. Cells were pooled from SJL/J mice in each group (n=5) and run in triplicate for quantification and stimulation procedures. Each bar represents mean percentage for every marker per group. (D) Further the activation of $CD11c^{+}$ DCs was analyzed within the peripheral blood. (E) Cells from peripheral blood of SJL/J mice from EAE+vehicle and EAE+CLEC12A antibody treatment were stained for CD11c, CD11b and CCR2 markers to plot CCR2 expression on DCs and monocytes. Bar graph represents comparative GMFI of CCR2 expression for group analyzed (n=5) with standard error bars. *$P<0.05$**.**

Supplementary Figure 8. Potential mechanisms of action of anti-CLEC12A antibody binding on DCs and ligand identification. ***A.*** This model depicts ligand binding to the CLEC12A receptor thereby leading to the downstream events of DC migration. The mechanism of action of antibody binding to the receptor can involve a model depicted in ***B.*** where 1. The antibody binding causes receptor neutralization making it unavailable to bind to other ligands. Here the SHP domains do not get phosphorylated. 2. Binding of anti–CLEC12A antibody leads to the internalization and activation of the CLEC12A receptor resulting in phosphorylation of SHP thereby triggering actin polymerization events subsequently leading to anti-inflammatory responses.

Supplementary Figure 1

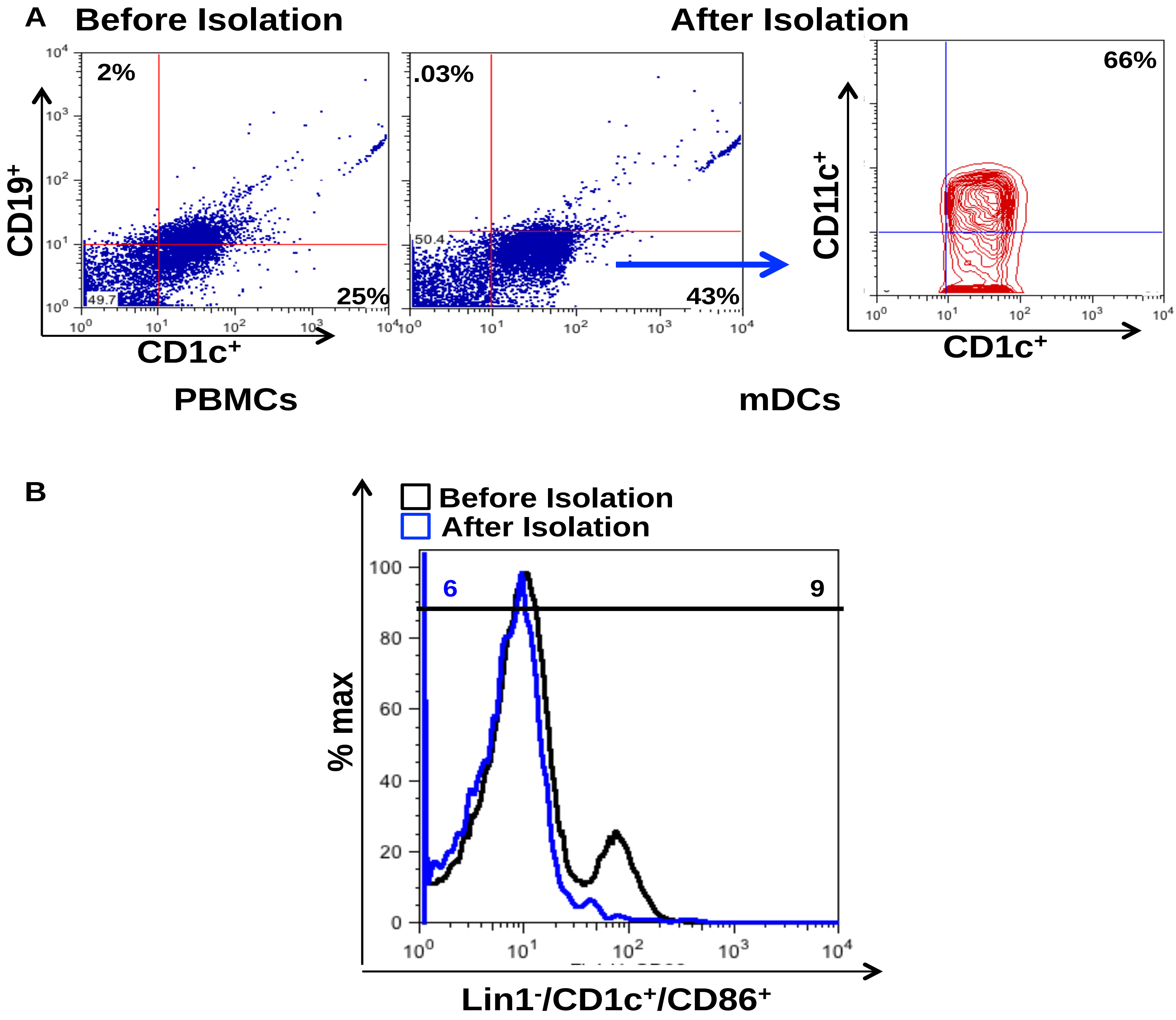

Supplementary Figure 2

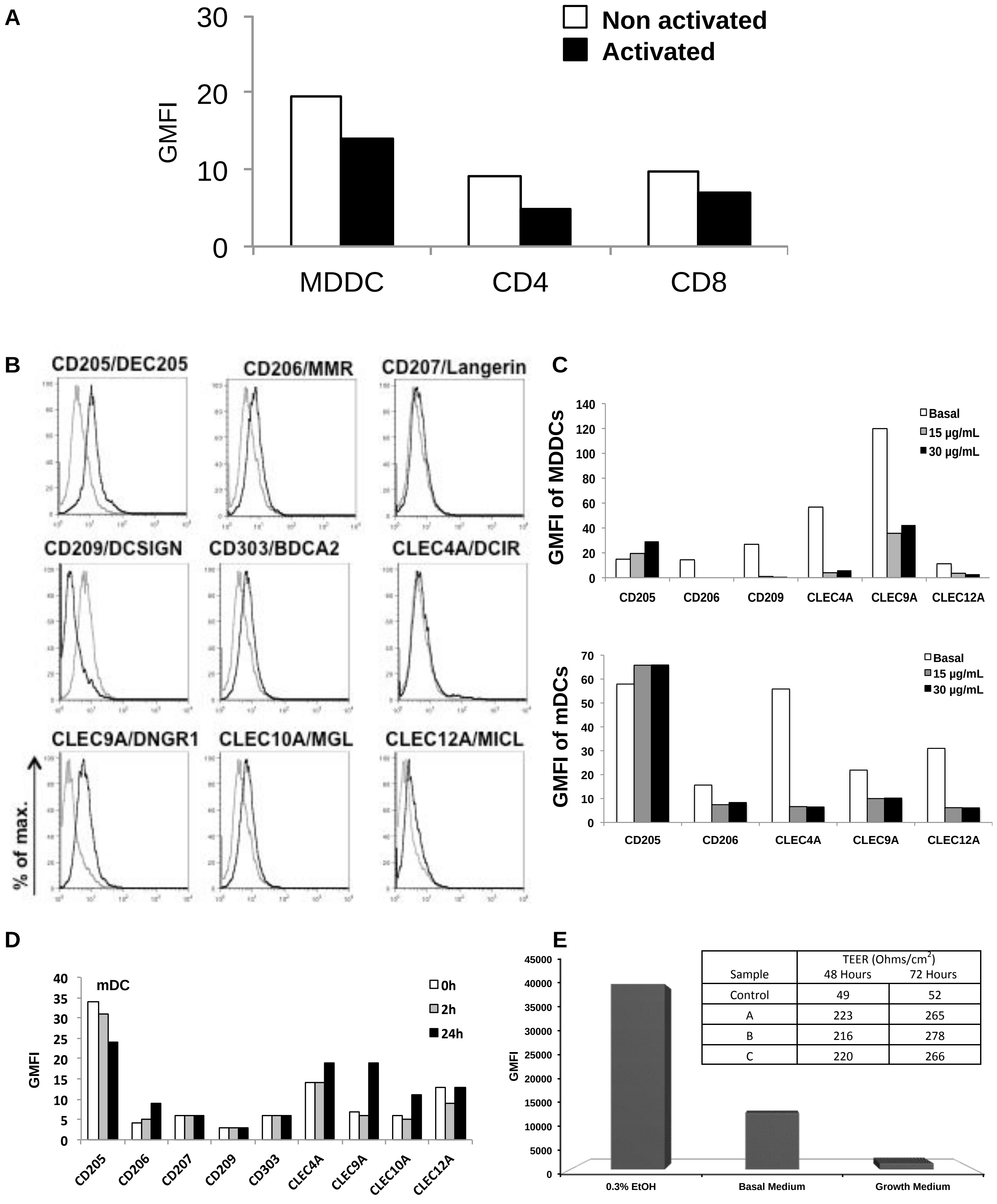

Sample	TEER (Ohms/cm^2) 48 Hours	72 Hours
Control	49	52
A	223	265
B	216	278
C	220	266

Supplementary Figure 3

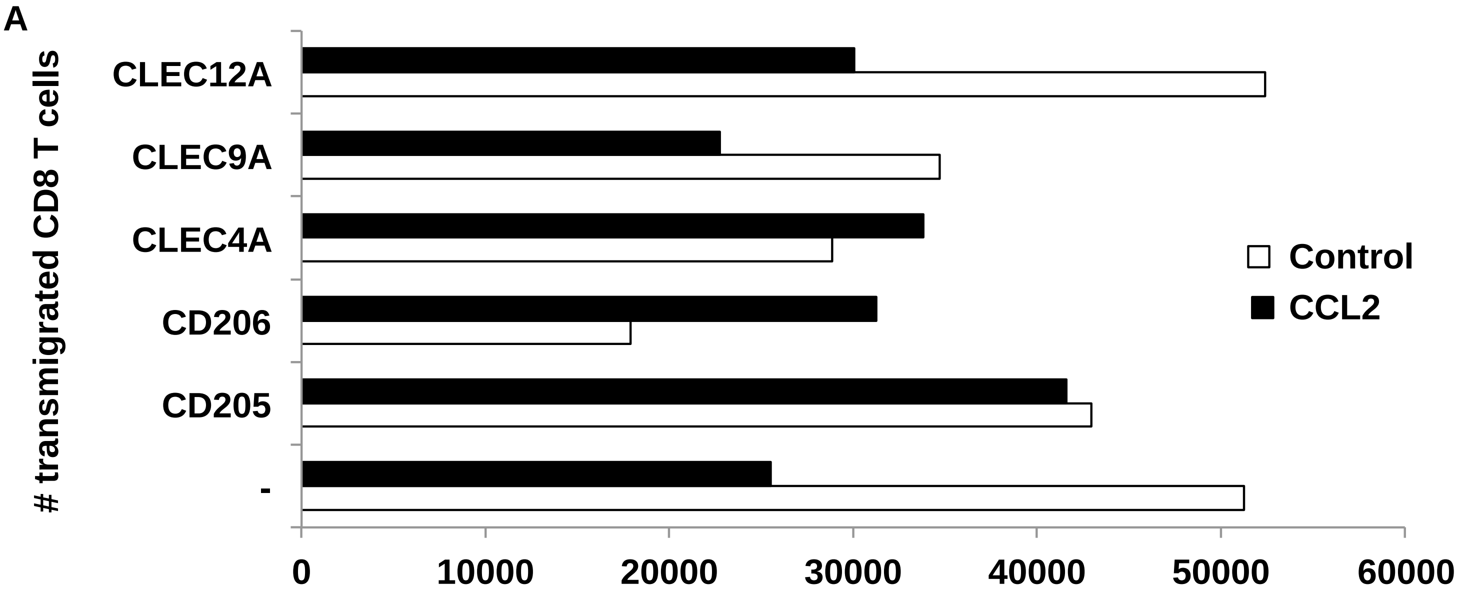

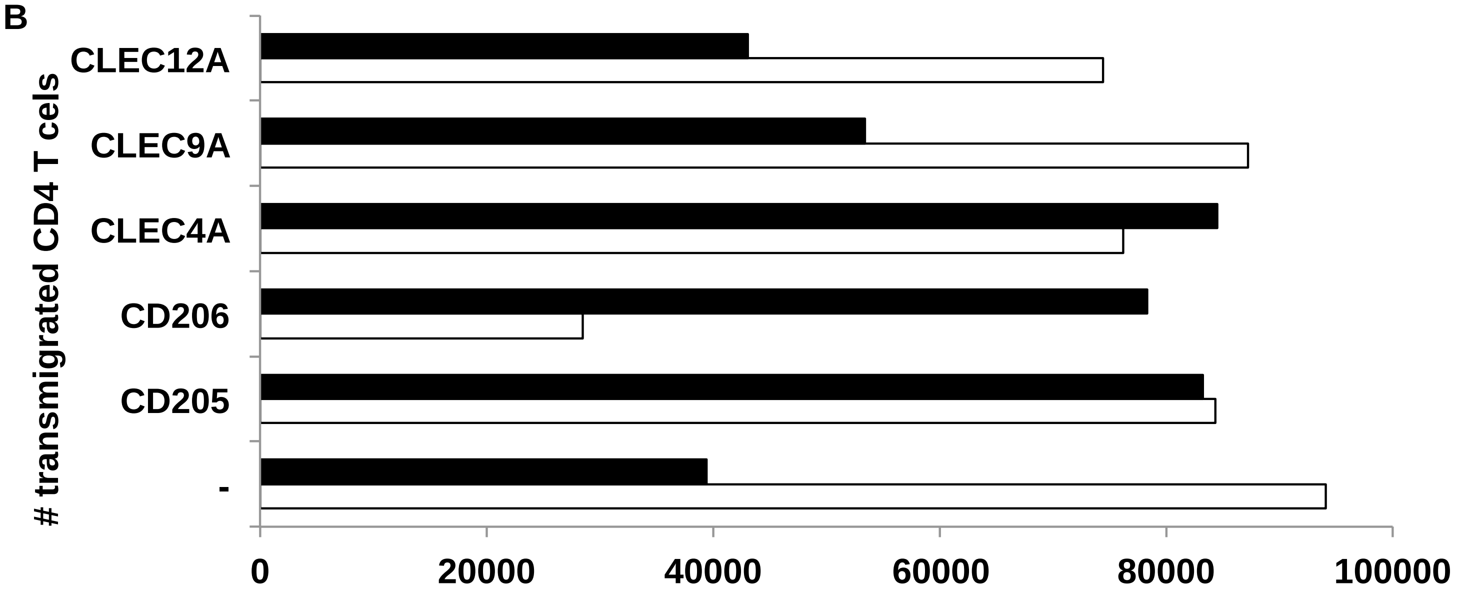

Supplementary Figure 4

A

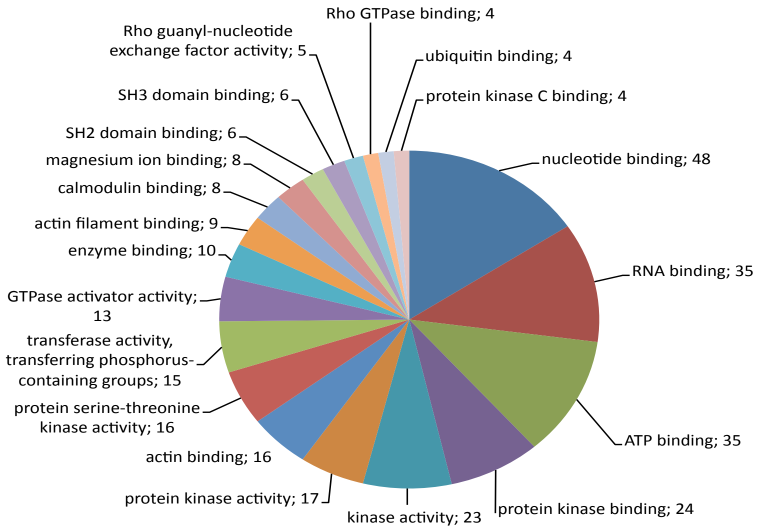

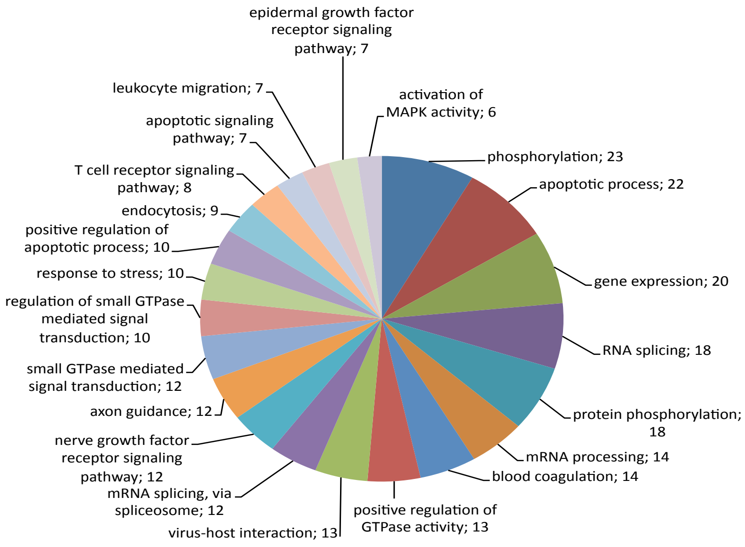

Supplementary Figure 4

B

Sequence: MCDFGISGYLVDSVAK, M1-Oxidation (15.99492 Da), C2-Carbamidomethyl (57.02146 Da), S13-Phospho (79.96633 Da)

Charge: +2, Monoisotopic m/z: 929.39520 Da (+1.59 mmu/+1.71 ppm), MH+: 1857.78313 Da, RT: 55.83 min, Identified with: SEQUEST (v1.20); XCorr:5.13, Ions matched by search engine: 36/59

Fragment match tolerance used for search: 0.8 Da

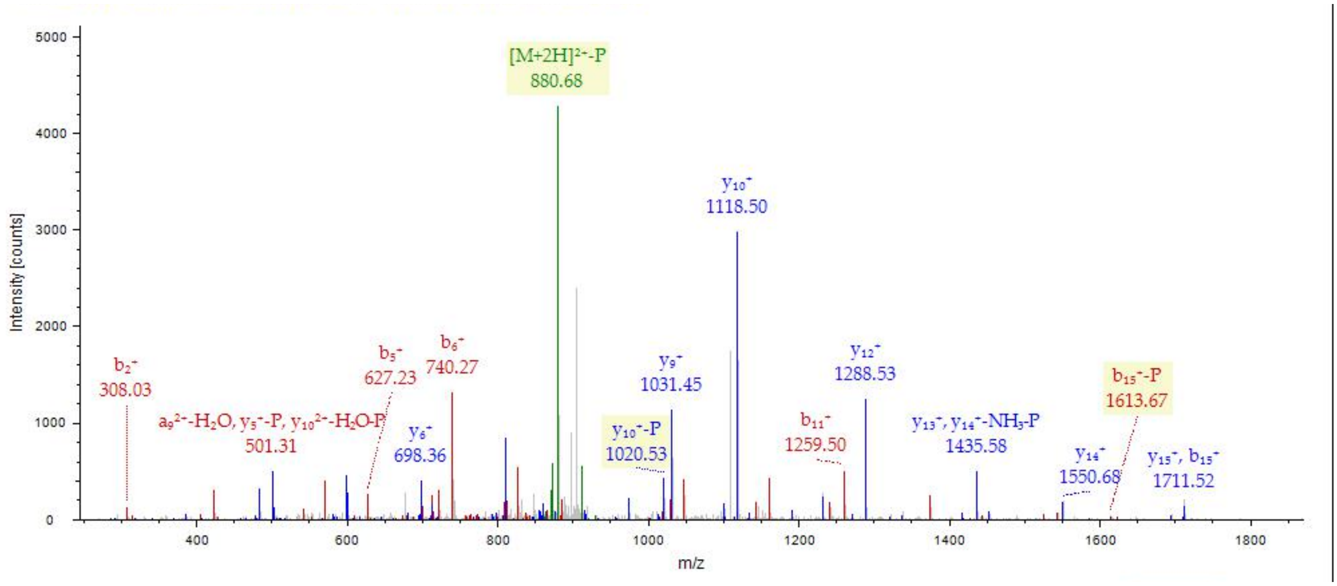

C

Sequence: LPSSPVYEDAASFK, S4-Phospho (79.96633 Da)

Charge: +2, Monoisotopic m/z: 795.85791 Da (-0.12 mmu/-0.16 ppm), MH+: 1590.70854 Da, RT: 48.10 min, Identified with: SEQUEST (v1.20); XCorr:3.24, Ions matched by search engine: 23/51

Fragment match tolerance used for search: 0.8 Da

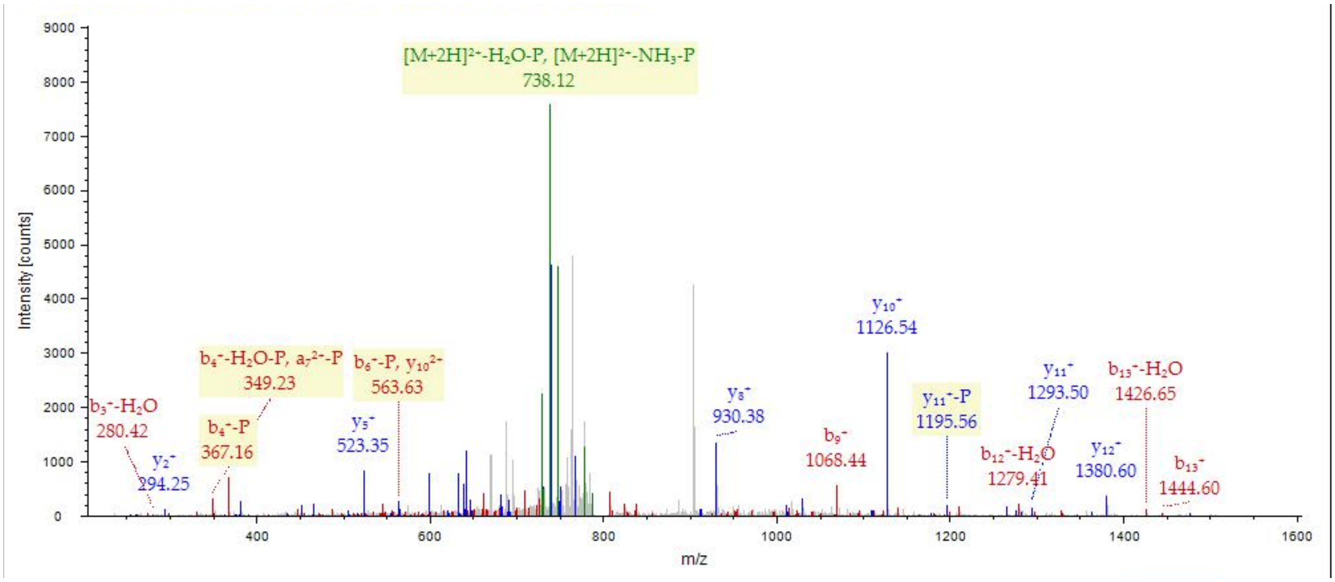

Supplementary Figure 5

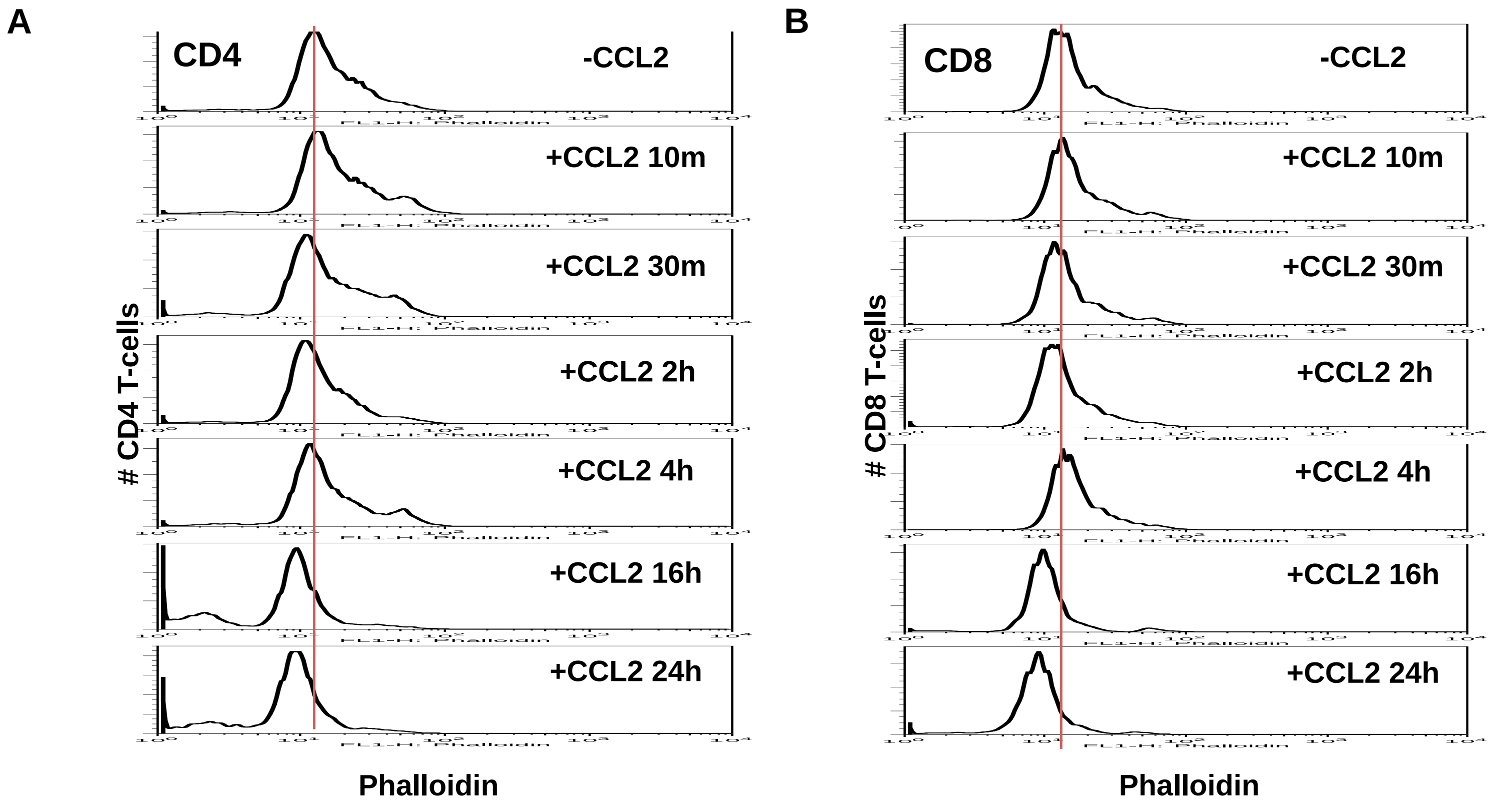

Supplementary Figure 6

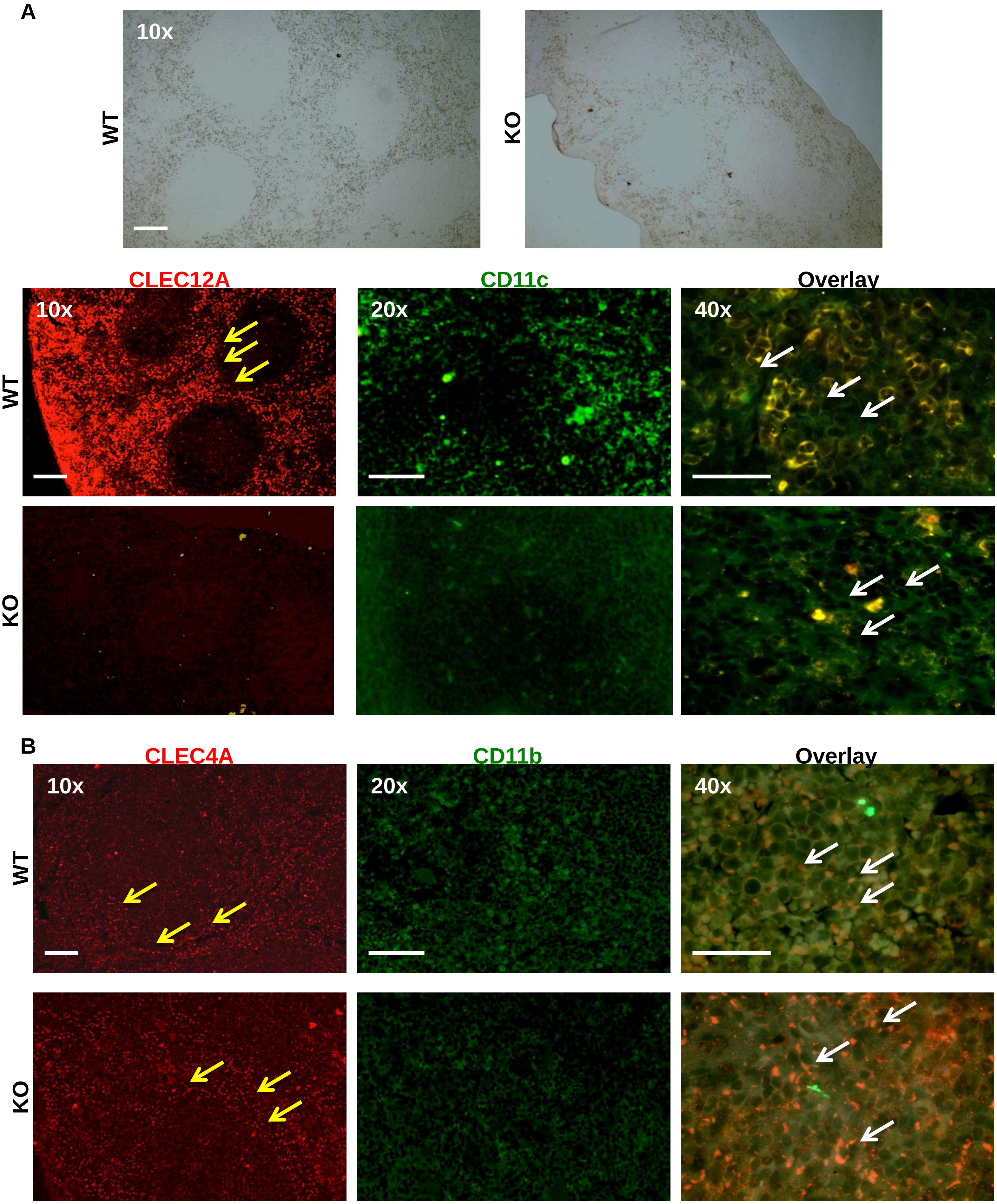

Supplementary Figure 7

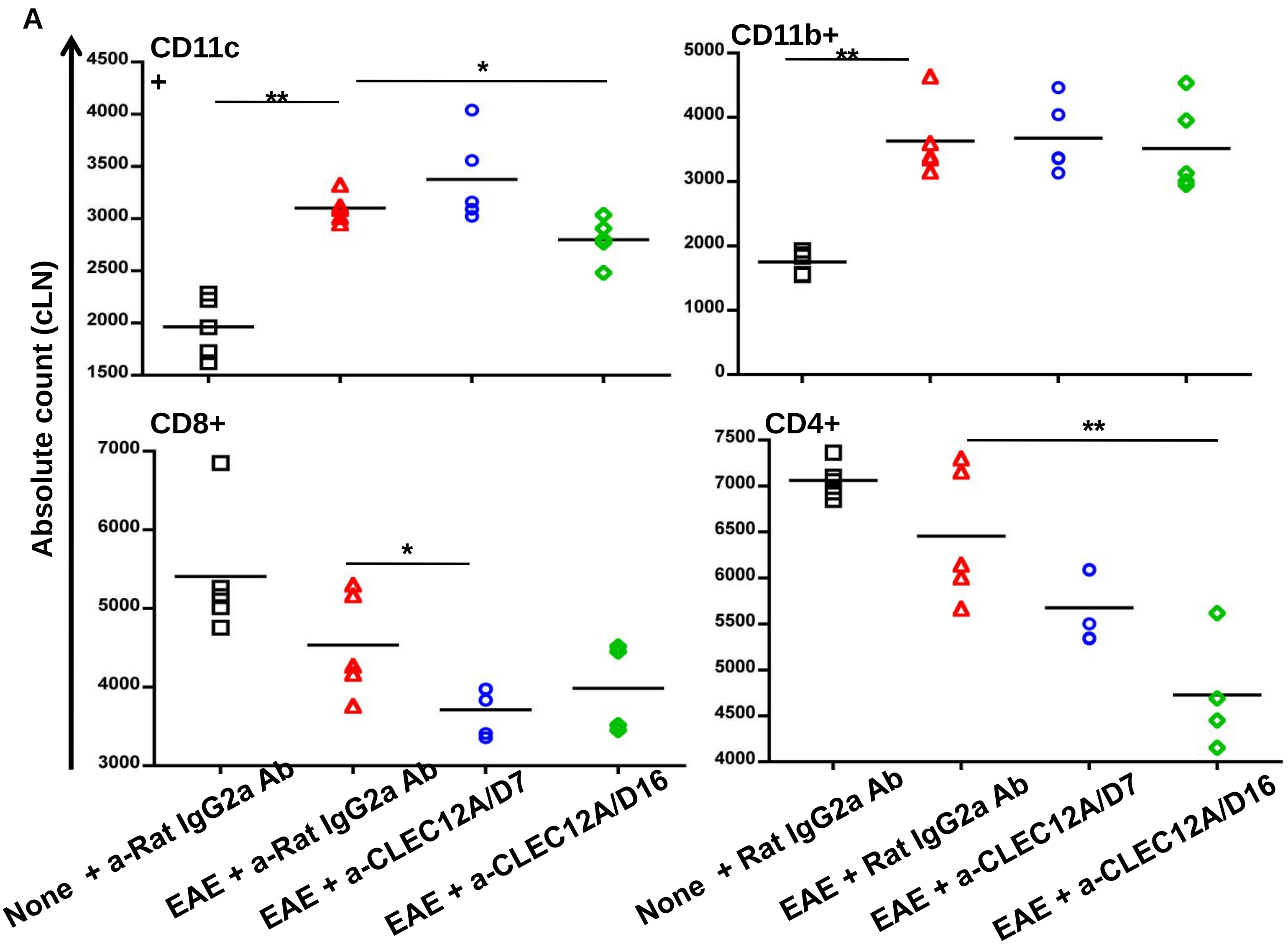

Supplementary Figure 7

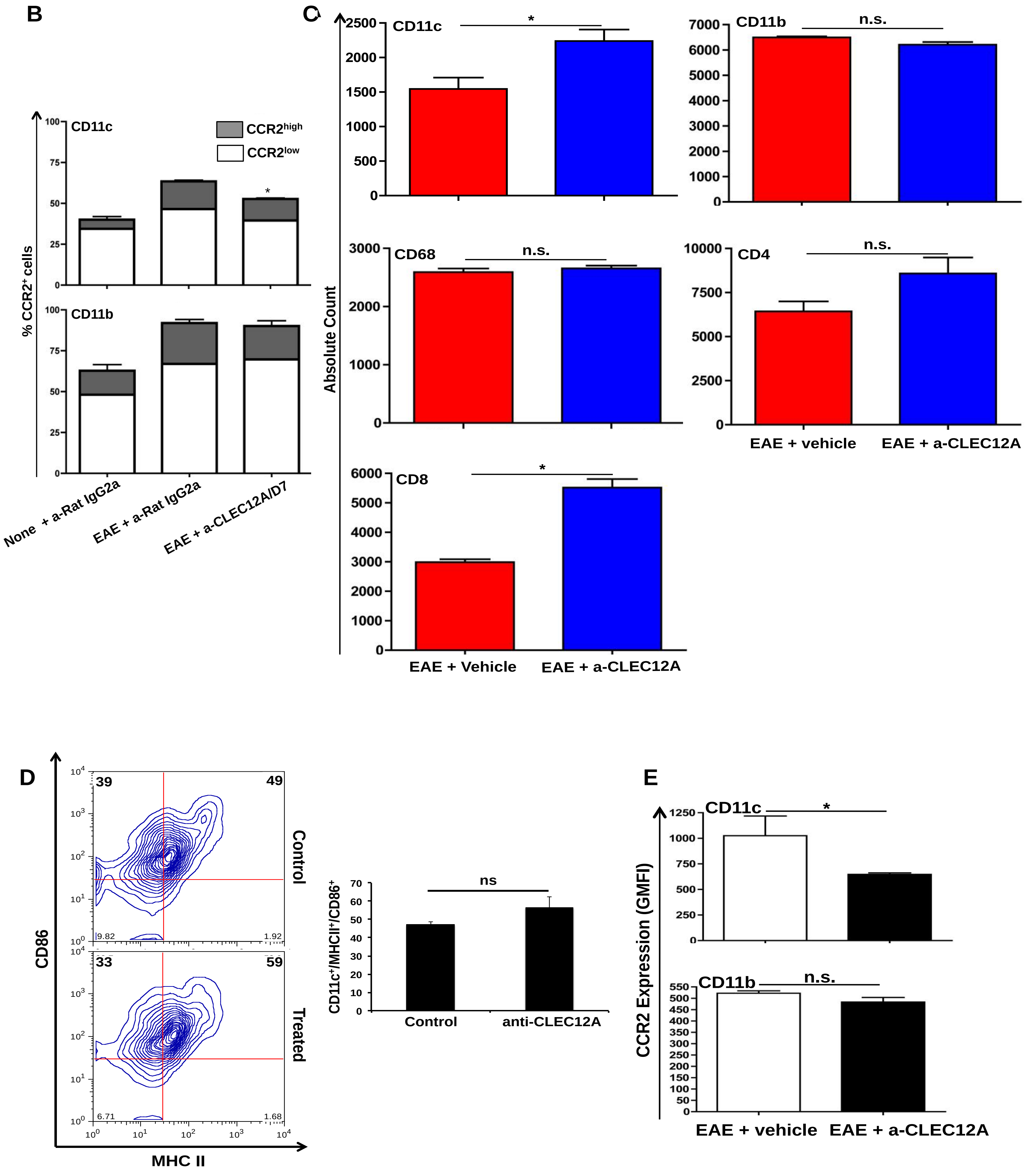

Supplementary Figure 8

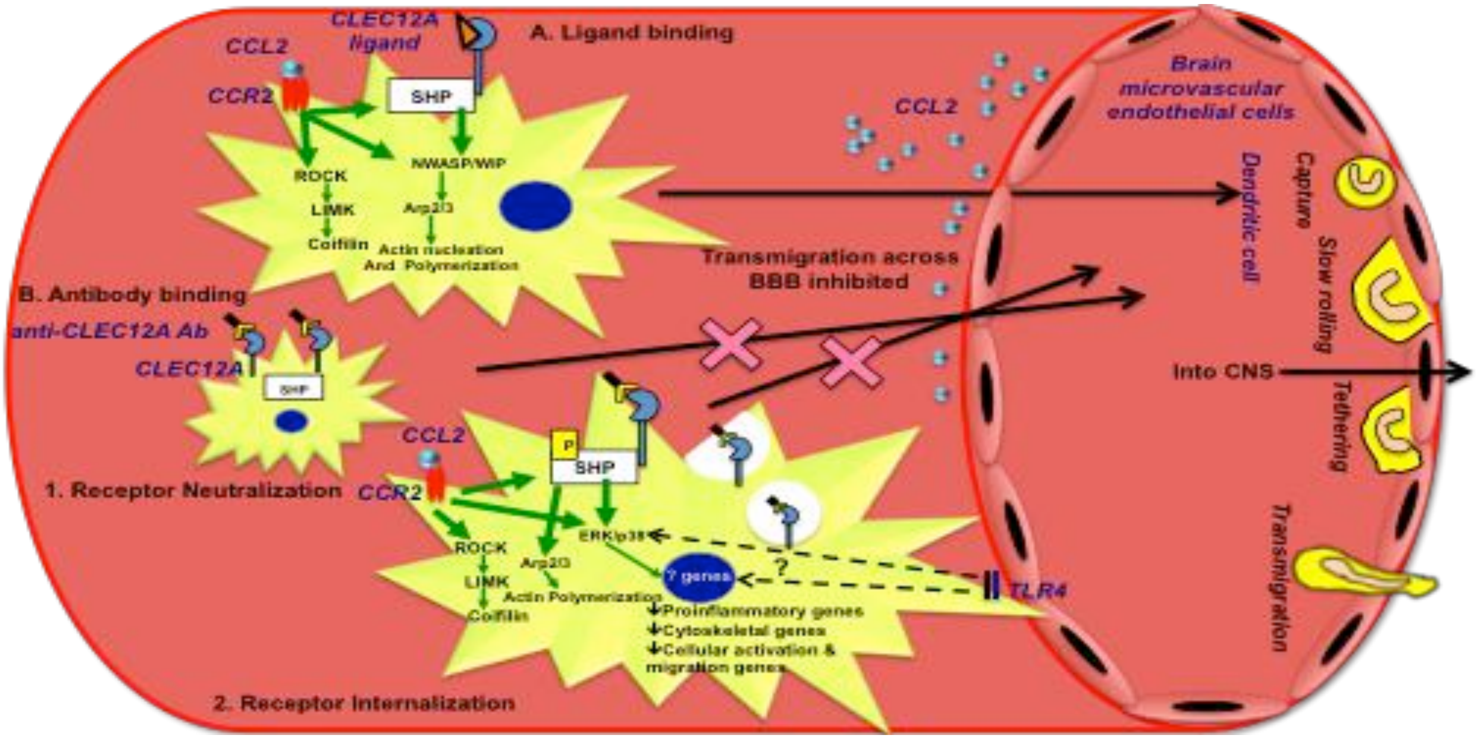

Table S1. Phosphopeptides found only present in CCL2 treatment sample.

Protein Description	Peptides	Modifications
Absent in melanoma 1 protein	DTCVQSPISSFPCTDLK	C3(Carbamidomethyl); S6(Phospho); C13(Carbamidomethyl)
Anion exchange protein 2	ALTQPSPVSTPSSVQFFLQEDDSADR	S6(Phospho)
Anoctamin-6	MIEAVDNNLRPKSE	S13(Phospho)
Apolipoprotein B receptor	LRSWEQEEEEEVR	S3(Phospho)
Arginine-glutamic acid dipeptide repeats protein	EKVASDTEEADR	S5(Phospho)
BMP-2-inducible protein kinase	RDSQSSNEFLTISDSK	S3(Phospho)
BSD domain-containing protein 1	KGSSTDISEDWEK	T5(Phospho)
Cerebellar degeneration-related protein 2	SSSETILSSLAGSDIVK	S3(Phospho)
CREB-regulated transcription coactivator 3	SNPSIQATLNK	S1(Phospho)
Crk-like protein	DSSTCPGDYVLSVSENSR	S2(Phospho); C5(Carbamidomethyl)
C-X-C chemokine receptor type 5	RSSLSESENATSLTTF	S3(Phospho)
Cyclin-L2	GLLPGGTQVLDGTSGFSPAPK	S17(Phospho)
Cytosolic phospholipase A2	HIVSNDSSDSDDESHEPK	S10(Phospho)
DNA-binding protein A	SVGDGETVEFDVVEGEK	S1(Phospho)
Dual specificity mitogen-activated protein kinase kinase 3	MCDFGISGYLVDSVAK	M1(Oxidation); C2(Carbamidomethyl); S13(Phospho)
Dual specificity mitogen-activated protein kinase kinase 6	MCDFGISGYLVDSVAK	C2(Carbamidomethyl); S13(Phospho)
E3 ubiquitin-protein ligase RBBP6	LEVTEIVKPSPK	S10(Phospho)
Emerin	RLSPPSSSAASSYSFSDLNSTR	S3(Phospho)
Epsin-1	GSLAEAVGSPPPAATPTPTPPTR	S9(Phospho)
FH1/FH2 domain-containing protein 1	TPQSPAPCVLLR	S4(Phospho); C8(Carbamidomethyl)
G-protein-signaling modulator 1	APSSDEECFFDLLTK	S3(Phospho); C8(Carbamidomethyl)
HLA class I histocompatibility antigen, Cw-12 alpha chain	GGSCSQAASSNSAQGSDESLIACK	C4(Carbamidomethyl); S16(Phospho); C23(Carbamidomethyl)
HLA class I histocompatibility antigen, Cw-14 alpha chain	GGSCSQAASSNSAQGSDESLIACK	C4(Carbamidomethyl); S16(Phospho); C23(Carbamidomethyl)
HLA class I histocompatibility antigen, Cw-15 alpha chain	GGSCSQAASSNSAQGSDESLIACK	C4(Carbamidomethyl); S16(Phospho); C23(Carbamidomethyl)
HLA class I histocompatibility antigen, Cw-16 alpha chain	GGSCSQAASSNSAQGSDESLIACK	C4(Carbamidomethyl); S16(Phospho); C23(Carbamidomethyl)
HLA class I histocompatibility antigen, Cw-17 alpha chain	GGSCSQAASSNSAQGSDESLIACK	C4(Carbamidomethyl); S16(Phospho); C23(Carbamidomethyl)
HLA class I histocompatibility antigen, Cw-18 alpha chain	GGSCSQAASSNSAQGSDESLIACK	C4(Carbamidomethyl); S16(Phospho); C23(Carbamidomethyl)
HLA class I histocompatibility antigen, Cw-3 alpha chain	GGSCSQAASSNSAQGSDESLIACK	C4(Carbamidomethyl); S16(Phospho); C23(Carbamidomethyl)
HLA class I histocompatibility antigen, Cw-4 alpha chain	GGSCSQAASSNSAQGSDESLIACK	C4(Carbamidomethyl); S16(Phospho); C23(Carbamidomethyl)
HLA class I histocompatibility antigen, Cw-5 alpha chain	GGSCSQAASSNSAQGSDESLIACK	C4(Carbamidomethyl); S16(Phospho); C23(Carbamidomethyl)
HLA class I histocompatibility antigen, Cw-6 alpha chain	GGSCSQAASSNSAQGSDESLIACK	C4(Carbamidomethyl); S16(Phospho); C23(Carbamidomethyl)
HLA class I histocompatibility antigen, Cw-8 alpha chain	GGSCSQAASSNSAQGSDESLIACK	C4(Carbamidomethyl); S16(Phospho); C23(Carbamidomethyl)
Integrator complex subunit 3	EKFPEFCSSPSPPVEVK	C7(Carbamidomethyl); S11(Phospho)
Kinesin light chain 1	SRESLNVDVVK	S1(Phospho); S4(Phospho)
Kinesin-like protein KIF13A	TGSCSELDACPSK	S3(Phospho); C4(Carbamidomethyl); C10(Carbamidomethyl)
La-related protein 4B	TLSADASVNTLPVVVSR	S3(Phospho)
Leucine-rich repeat flightless-interacting protein 2	RGSGDTSSLIDPDTSLSELR	S3(Phospho)
MAP7 domain-containing protein 1	RSSQPSPTAVPASDSPPTK	S3(Phospho)
Mediator of DNA damage checkpoint protein 1	LLLAEDSEEEVDFLSER	S7(Phospho)
Mediator of RNA polymerase II transcription subunit 24	LLSSNEDDANILSSPTDR	S3(Phospho)
Melanoma-associated antigen D2	HLDGEEDGSSDQSQASGTTGGR	S9(Phospho)
Methyl-CpG-binding protein 2	AETSEGSGSAPAVPEASASPK	S19(Phospho)
Microtubule-associated protein 1S	AVPMAPAPASPGSSNDSSAR	M4(Oxidation); S10(Phospho)
Mitochondrial antiviral-signaling protein	GPVSPSVSFQPLAR	S4(Phospho)
MLN64 N-terminal domain homolog	LLIVQDASER	S8(Phospho)
Myosin-Va	TSSIADEGTYTLDSILR	S3(Phospho)
Nuclear factor of activated T-cells, cytoplasmic 3	KTSEDQAAILPGK	S3(Phospho)
Nuclear pore complex protein Nup214	SPGSTPTTPTSSQAPQK	S4(Phospho); T8(Phospho)
Nucleolar RNA helicase 2	NEEPSEEEIDAPKPK	S5(Phospho)
Nucleoporin NUP188 homolog	GAPSSPATGVLPSPQGK	S5(Phospho)
Peptidyl-prolyl cis-trans isomerase G	KFDHESSPGTDEDKSG	S15(Phospho)
PERQ amino acid-rich with GYF domain-containing protein 2	ALSSGGSITSPPLSPALPK	S10(Phospho); S14(Phospho)
Phosphorylase b kinase regulatory subunit alpha, liver isoform	SLNLVDSPQPLLEK	S1(Phospho)
Plasminogen activator inhibitor 1 RNA-binding protein	TDKSSASAPDVDDPEAFPALA	S4(Phospho)
Pleckstrin homology domain-containing family A member 2	SQSYIPTSGCR	S3(Phospho); C10(Carbamidomethyl)
Pre-mRNA 3'-end-processing factor FIP1	DHSPTPSVFNSDEER	S3(Phospho)
Programmed cell death protein 5	KVMDSDEDDDY	S5(Phospho)
Protein FAM122B	RIDFTPVSPAPSPTR	S8(Phospho); S12(Phospho)
Protein FAM65A	FSTYSQSPPDTPSLR	S7(Phospho)
Protein FAM65B	SQSFAGFSGLQER	S3(Phospho)
Protein PAT1 homolog 1	RSTSPIIGSPPVR	S2(Phospho); S4(Phospho)
Protein RIC1 homolog	SISLSQSAENVPASK	S3(Phospho)
Raftlin	GDHASLENEKPGTGDVCSAPAGR	S5(Phospho); C17(Carbamidomethyl)
Rap1 GTPase-activating protein 1	AAGISLIVPGKSPTR	S12(Phospho)
Ras GTPase-activating-like protein IQGAP1	SKSVKEDSNLTLQEK	S3(Phospho)
Receptor-interacting serine/threonine-protein kinase 2	SPSLNLLQNK	S3(Phospho)
Ribonuclease P protein subunit p30	KPRPSEGDEDCLPASK	S5(Phospho); C11(Carbamidomethyl)
Ribonucleoprotein PTB-binding 1	EALGLGPPAAQLTPPPAPVGLR	T13(Phospho)
Ribosomal RNA-processing protein 8	QGPPCSDSEEEVER	C5(Carbamidomethyl); S8(Phospho)
RING finger protein 31	RLSAPLPSSCGDPEK	S3(Phospho); C10(Carbamidomethyl)
RNA-binding protein NOB1	KDDSDDDGGGWITPSNIK	S4(Phospho)
Run domain Beclin-1 interacting and cystein-rich containing protein	RPSEGQSLISYLSEQDFGSCADLEK	S3(Phospho); C20(Carbamidomethyl)
Serine/arginine-rich splicing factor 6	SNSPLPVPPSK	S3(Phospho)
Serine/threonine-protein kinase SIK3	RASDGGANIQLHAQQLLK	S3(Phospho)
	TWCGSPPYAAPELFEGK	T1(Phospho); C3(Carbamidomethyl)
Signal-induced proliferation-associated protein 1	TEFLHSQNSLSPR	S11(Phospho)
Sister chromatid cohesion protein PDS5 homolog B	AESPESSAIESTQSTPQK	S3(Phospho)
Smith-Magenis syndrome chromosomal region candidate gene 8 protein	SDSQASLTVPLSPQVVR	S3(Phospho); S6(Phospho)
Sodium/hydrogen exchanger 7	SSSEEVLER	S2(Phospho)
Sodium/myo-inositol cotransporter	SEDSIKGLQPEDVNLLVTCR	S1(Phospho); C19(Carbamidomethyl)
Solute carrier organic anion transporter family member 4C1	GIENLAFVPSSPDILR	S11(Phospho)
Sortilin	SGYHDDSDEDLLE	S7(Phospho)
Src substrate cortactin	LPSSPVYEDAASFK	S4(Phospho)
Sulfide:quinone oxidoreductase, mitochondrial	YPNVFGIGDCTNLPTSK	C10(Carbamidomethyl); T15(Phospho)
Suppressor of fused homolog	KDSLESDSSTAIIPHELIR	S3(Phospho)
Thymosin beta-4	TETQEKNPLPSK	T3(Phospho)
Thymosin beta-4-like protein 3	TETQEKNPLPSK	T3(Phospho)
Trafficking protein particle complex subunit 10	RQESSSSLEMPSGVALEEGAHVLR	S4(Phospho); S7(Phospho)
Transcription elongation factor SPT6	TRTPASINATPANINLADLTR	T3(Phospho); S6(Phospho)
Transcription factor EB	RSSFSMEEGDVL	S3(Phospho); M6(Oxidation)
Transgelin-2	NFSDNQLQEGK	S3(Phospho)
Transmembrane and coiled-coil domains protein 1	FGSADNIPNLK	S3(Phospho)
Transmembrane protein C9orf5	GESAPTLSTSPSPSSPSPTSPSPTLGR	S22(Phospho)
Ubiquitin-associated protein 1	GGSGSVLQDEEVLASLER	S3(Phospho)
Uncharacterized protein C18orf25	RDSSESQLASTESDKPTTGR	S3(Phospho)
Uncharacterized protein C9orf78	RRGDSESEEDEQDSEEVR	S5(Phospho); S7(Phospho)
Vesicle transport protein SFT2A	VLSGQDDEEQGLTAQVLDASSLSFNTR	S3(Phospho)
Vigilin	VATLNSEEESDPPTYK	S6(Phospho)
Y-box-binding protein 2	SVGDGETVEFDVVEGEK	S1(Phospho)
Zinc finger CCHC domain-containing protein 8	KSEAGHASSPDSEVTSLCQK	S9(Phospho); C18(Carbamidomethyl)
Zinc finger FYVE domain-containing protein 26	SPSAEFSPAAPPGISSIHSPSLR	S1(Phospho)
Zinc finger RNA-binding protein	RRDSDGVDGFEAEGK	S4(Phospho)

Table S2. Phosphopeptides found only present

Accession ID	Protein Description	Peptide	Modifications
P63104	14-3-3 protein zeta/delta	TAFDEAIAELDTLSEESYK	S14(Phospho)
Q99460	26S proteasome non-ATPase regulatory subunit 1	TSSAFVGKTPEASPEPK	T9(Phospho); S13(Phospho)
P12694	2-oxoisovalerate dehydrogenase subunit alpha, mitochondrial	IGHHSTSDDSSAYR	S5(Phospho)
Q96D46	60S ribosomal export protein NMD3	DSAIPVESDTDDEGAPR	T10(Phospho)
P53999	Activated RNA polymerase II transcriptional coactivator p15	ELVSSSSSGSDSDSEVDKK	S12(Phospho)
Q02952	A-kinase anchor protein 12	GLAEVQQDGEAEEGATSDGEK	S17(Phospho)
O43823	A-kinase anchor protein 8	VDSEGDFSENDDAAGDFR	S8(Phospho)
Q53LP3	Ankyrin repeat domain-containing protein 57	DLVMGSSPQLK	M4(Oxidation); S7(Phospho)
P78410	Butyrophilin subfamily 3 member A2	EITALSSEIESEQEMK	S7(Phospho); S11(Phospho); M15(Oxidation)
P22612	cAMP-dependent protein kinase catalytic subunit gamma	TWTLCGTPEYLAPEIILSK	T3(Phospho); C5(Carbamidomethyl)
Q9NZN8	CCR4-NOT transcription complex subunit 2	TNSMSSSGLGSPNR	M4(Oxidation); S11(Phospho)
Q9Y3E7	Charged multivesicular body protein 3	VTDALPEPEPPGAMAASEDEEEEEEALEAMQSR	M14(Oxidation); S17(Phospho); M30(Oxidation)
Q8WUX9	Charged multivesicular body protein 7	ISDAELEAELEK	S2(Phospho)
P49585	Choline-phosphate cytidylyltransferase A	AAAYDISEDEED	S7(Phospho)
Q15846	Clusterin-like protein 1	IPLEESAESSNFIGYVVAK	S6(Phospho); S10(Phospho)
Q8IV53	DENN domain-containing protein 1C	KLPEPEPQPLSLPSLQNASSLDATSSSK	S11(Phospho)
P49959	Double-strand break repair protein MRE11A	NYSEVIEVDESDVEEDIFPTTSK	S11(Phospho)
Q9UJU6	Drebrin-like protein	YQEQGGEASPQR	S9(Phospho)
P49792	E3 SUMO-protein ligase RanBP2	VGEDEDGSDEEVVHNEDIHFEPIVSLPEVEVK	S8(Phospho)
Q8WZ73	E3 ubiquitin-protein ligase rififylin	ASLSDLTDLEDIEGLTVR	S2(Phospho)
Q969K3	E3 ubiquitin-protein ligase RNF34	ASLSDLSSLDDVEGMSVR	S4(Phospho); M15(Oxidation)
Q14152	Eukaryotic translation initiation factor 3 subunit A	LGDSSLSR	S5(Phospho)
Q01167	Forkhead box protein K2	EGSPAPLEPEPGAAQPK	S3(Phospho)
O60763	General vesicular transport factor p115	DLGHPVEEEDELESGDQEDEDDESEDPGK	S14(Phospho)
Q9NQX3	Gephyrin	EVHDELEDLPSPPPPLSPPPTTSPHK	S11(Phospho); S17(Phospho)
Q3V6T2	Girdin	DTTSFEDISPQGVSDDSSTGSR	S4(Phospho)
Q86X53	Glutamate-rich protein 1	EEDGADASEEDLTR	S8(Phospho)
Q9HCN4	GPN-loop GTPase 1	GTLDEEDEEADSDTDDIDHR	S12(Phospho)
Q15477	Helicase SKI2W	ASSLEDLVLK	S3(Phospho)
P17096	High mobility group protein HMG-I/HMG-Y	EEEEGISQESSEEEQ	S10(Phospho)
Q9UPR0	Inactive phospholipase C-like protein 2	LSSNCSGVEGDVTDEDEGAEMSQR	C5(Carbamidomethyl); T13(Phospho); M21(Oxidation)
O14920	Inhibitor of nuclear factor kappa-B kinase subunit beta	LSQPGQLMSQPSTASNSLPEPAK	M8(Oxidation); S17(Phospho)
		GPVSGSPDSMNASR	S6(Phospho); M10(Oxidation)
Q8IU81	Interferon regulatory factor 2-binding protein 1	KASPEPEGEAAGK	S3(Phospho)
Q86V48	Leucine zipper protein 1	EKPDSDDDLDIASLVTAK	S5(Phospho)
Q86X29	Lipolysis-stimulated lipoprotein receptor	SVDALDDLTPPSTAESGSR	S1(Phospho)
P09603	Macrophage colony-stimulating factor 1	ADSPLEQPEGSPLTQDDR	S3(Phospho)
P40925	Malate dehydrogenase, cytoplasmic	KLSSAMSAAK	S3(Phospho); M6(Oxidation)
O15427	Monocarboxylate transporter 4	NGEVVHTPETSV	S11(Phospho)
Q9NYA4	Myotubularin-related protein 4	SMDDLLSACDTSSPLTR	S1(Phospho); M2(Oxidation); C9(Carbamidomethyl)
P23497	Nuclear autoantigen Sp-100	SEPVINNDNPLESNDEK	S13(Phospho)
Q01804	OTU domain-containing protein 4	GELDLSLENLDLSK	S6(Phospho)
Q9BZF1	Oxysterol-binding protein-related protein 8	SDKENDQEHDESDNEVMGK	S12(Phospho); M17(Oxidation)
O75475	PC4 and SFRS1-interacting protein	TGVTSTSDSEEEGDDQEGEK	S9(Phospho)
Q13610	Periodic tryptophan protein 1 homolog	LQEEGGGSDEEETGSPSEDGMQSAR	S8(Phospho); M21(Oxidation)
Q8TCU6	Phosphatidylinositol 3,4,5-trisphosphate-dependent Rac exchanger 1 protein	SNSSYLGSDEMGSGDELPCDMR	M11(Oxidation); S13(Phospho); C19(Carbamidomethyl); M21(Oxidation)
Q9UBF8	Phosphatidylinositol 4-kinase beta	SVENLPECGITHEQR	S1(Phospho); C8(Carbamidomethyl)
Q9H307	Pinin	EIAIVHSDAEK	S7(Phospho)
Q9UKK3	Poly [ADP-ribose] polymerase 4	TTPVDLCLLEESVGSLEGSR	T1(Phospho); C7(Carbamidomethyl)
Q13563	Polycystin-2	SLDDSEEDDDEDSGHSSR	S5(Phospho)
P42694	Probable helicase with zinc finger domain	RISSSSVQPCSEEVSTPQDSLAQCK	S4(Phospho); C10(Carbamidomethyl); C24(Carbamidomethyl)
Q93008	Probable ubiquitin carboxyl-terminal hydrolase FAF-X	NGILAIEGTGSDVDDDMSGDEK	M17(Oxidation); S18(Phospho)
Q8NBJ5	Procollagen galactosyltransferase 1	NSDVLQSPLDSAARDEL	S11(Phospho)
Q9NZQ7	Programmed cell death 1 ligand 1	KQSDTHLEET	S3(Phospho)
O43586	Proline-serine-threonine phosphatase-interacting protein 1	LTLEGCSIDADIDSFIQAK	C6(Carbamidomethyl); S7(Phospho)
Q13158	Protein FADD	SGAMSPMSWNSDASTSEAS	M4(Oxidation); S5(Phospho); M7(Oxidation)
Q04759	Protein kinase C theta type	TNTFCGTPDYIAPEILLGQK	T1(Phospho); C5(Carbamidomethyl)
Q86UE4	Protein LYRIC	SQEPIPDDQKVSDDDKEK	S12(Phospho)
Q9UGV2	Protein NDRG3	THSTSSSLGSGESPFSR	S3(Phospho); S6(Phospho)
P41236	Protein phosphatase inhibitor 2	IQEQESSGEEDSDLSPEER	S6(Phospho); S7(Phospho)
P06702	Protein S100-A9	MHEGDEGPGHHHKPGLGEGTP	M1(Oxidation); T20(Phospho)
Q9NVU7	Protein SDA1 homolog	YIEIDSDEEPR	S6(Phospho)
Q01105	Protein SET	LNEQASEEILK	S6(Phospho)
P12931	Proto-oncogene tyrosine-protein kinase Src	LIEDNEYTAR	Y7(Phospho)
Q58FF7	Putative heat shock protein HSP 90-beta-3	EISDDEAEEEK	S3(Phospho)
O60231	Putative pre-mRNA-splicing factor ATP-dependent RNA helicase DHX16	LLEDSEESSEETVSR	S8(Phospho)
Q6NXS1	Putative protein phosphatase inhibitor 2-like protein 3	IQEQESSGEEDSDLSPEER	S6(Phospho); S7(Phospho)
Q9BRQ0	Pygopus homolog 2	GGGTPDANSLAPPGK	T4(Phospho)
P31751	RAC-beta serine/threonine-protein kinase	YFDDEFTAQSITITPPDR	T14(Phospho)
Q8TEU7	Rap guanine nucleotide exchange factor 6	LPEGPVDSEDDEEEDEEIDR	S8(Phospho)
		LGDVTDADSEADENEQVSAV	S9(Phospho)
Q9HB90	Ras-related GTP-binding protein C	MSPNETLFLESTNK	M1(Oxidation); T12(Phospho)
Q9NQL2	Ras-related GTP-binding protein D	MSPNETLFLESTNK	M1(Oxidation); T12(Phospho)
Q6NUK4	Receptor expression-enhancing protein 3	DGDEKTDEEAEGPYSDNEMLTHK	T6(Phospho); M19(Oxidation)
O75582	Ribosomal protein S6 kinase alpha-5	DELDVSNFAEEFTEMDPTYSPAALPQSSEK	S6(Phospho); M15(Oxidation)
O15541	RING finger protein 113A	YGVYEDENYEVGSDDEEIPFK	S13(Phospho)
Q8N488	RING1 and YY1-binding protein	DKEISPSVTK	S5(Phospho)
Q15633	RISC-loading complex subunit TARBP2	SPPMELQPPVSPQQSECNPVGALQELVVQK	M4(Oxidation); S11(Phospho); C17(Carbamidomethyl)
Q9NWH9	SAFB-like transcription modulator	DGQDAIAQSPEK	S9(Phospho)
O95747	Serine/threonine-protein kinase OSR1	TEDGGWEWSDDEFDEESEEGK	S9(Phospho)
Q9BVS4	Serine/threonine-protein kinase RIO2	EGSEFSFSDGEVAEK	S8(Phospho)
P11831	Serum response factor	ALIQTCLNSPDSPPR	C6(Carbamidomethyl); S12(Phospho)
O14492	SH2B adapter protein 2	HSWVADIQGCVDPGDSEEDTELSCTR	C10(Carbamidomethyl); S16(Phospho); C24(Carbamidomethyl)
Q9Y336	Sialic acid-binding Ig-like lectin 9	GQEATDTEYSEIK	Y9(Phospho)
Q9Y6M7	Sodium bicarbonate cotransporter 3	MLQDDDDTVHLPFEGGSLLQIPVK	M1(Oxidation); T8(Phospho); S17(Phospho)
Q8TEQ0	Sorting nexin-29	GPVPGDLSQTSEDQSLSDFEISNR	S11(Phospho)
Q13813	Spectrin alpha chain, brain	LDPAQSASRENLLEEQGSIALR	S8(Phospho)
Q01082	Spectrin beta chain, brain 1	AQTLPTSVVTITSESSPGK	S16(Phospho)
Q8TF01	Splicing factor, arginine/serine-rich 18	SKFDSDEEEEDTENVEAASSGK	S5(Phospho)
O94864	STAGA complex 65 subunit gamma	TEESEPLPSCPGSPPLPDDLLPLDCK	C10(Carbamidomethyl); S13(Phospho); C25(Carbamidomethyl)
Q9UQE7	Structural maintenance of chromosomes protein 3	KGDVEGSQSQDEGEGSGESER	S9(Phospho)
Q9UBT2	SUMO-activating enzyme subunit 2	YLFNQLFGEEDADQEVSPDR	S17(Phospho)
O60264	SWI/SNF-related matrix-associated actin-dependent regulator of chromatin sub	GGPEGVAAQAVASAASAGPADAEMEEIFDDASP GK	M24(Oxidation); S32(Phospho)
Q92804	TATA-binding protein-associated factor 2N	TDADSESDNSDNNTIFVQGLGEGVSTDQVGEFF K	S7(Phospho)
Q9BUR4	Telomerase Cajal body protein 1	VFPEPTESGDEGEELGLPLLSTR	S8(Phospho)
Q969X1	Transmembrane BAX inhibitor motif-containing protein 1	AVSDSFGPGEWDDR	S3(Phospho)
O14683	Tumor protein p53-inducible protein 11	KHSQTDLVSR	S3(Phospho)
P06241	Tyrosine-protein kinase Fyn	LIEDNEYTAR	Y7(Phospho)
P07947	Tyrosine-protein kinase Yes	LIEDNEYTAR	Y7(Phospho)
P35236	Tyrosine-protein phosphatase non-receptor type 7	RGSNVALMLDVR	S3(Phospho); M8(Oxidation)
Q9Y385	Ubiquitin-conjugating enzyme E2 J1	RLSTSPDVIQGHQPR	S3(Phospho)
O95183	Vesicle-associated membrane protein 5	SDQLLDMSSTFNK	M7(Oxidation); S8(Phospho)
P18206	Vinculin	DPSASPGDAGEQAIR	S5(Phospho)

Table S3. Phophopeptides with unique phosphorylation site obtained from proteins identified in the CCL2 treatment sample.

Accession ID	Description	Peptides	Modifications
Q13442	28 kDa heat- and acid-stable phosphoprotein	KSLDSDESEDEEDDYQQK	S5(Phospho)
Q66PJ3	ADP-ribosylation factor-like protein 6-interacting protein 4	ASTAPGAEASPSPCITER	S10(Phospho); C14(Carbamidomethyl)
Q6ULP2	Aftiphilin	KFTNFQSPNIDPTEENDLDDSLSVK	S23(Phospho)
Q9UKA4	A-kinase anchor protein 11	YPSCESVTDEYAGHLIQILK	Y1(Phospho); C4(Carbamidomethyl)
Q9Y2D5	A-kinase anchor protein 2	DALGDSLQVPVSPSSTTSSR	S12(Phospho)
P35611	Alpha-adducin	AAVVTSPPPTTAPHK	S6(Phospho)
Q9UKV3	Apoptotic chromatin condensation inducer in the nucleus	KSSSISEEKGDSDDEKPR	S4(Phospho); S12(Phospho)
P53396	ATP-citrate synthase	AKPAMPQDSVPSPR	S12(Phospho)
Q9GZR7	ATP-dependent RNA helicase DDX24	AQAVSEEEEEEEGK	S5(Phospho)
P35613	Basigin	KPEDVLDDDDAGSAPLK	S13(Phospho)
Q9NYF8	Bcl-2-associated transcription factor 1	LKDLFDYSPPLHK	S8(Phospho)
Q9UBW5	Bridging integrator 2	TATVSSPLTSPTSPSTLSLK	S13(Phospho)
P53805	Calcipressin-1	QFLISPPASPPVGWK	S5(Phospho); S9(Phospho)
Q9UKA8	Calcipressin-3	QFLISPPASPPVGWK	S5(Phospho); S9(Phospho)
Q6JBY9	CapZ-interacting protein	VKSSPLIEK	S4(Phospho)
Q5SW79	Centrosomal protein of 170 kDa	SIKSDVPVYLK	S4(Phospho)
Q7Z401	C-myc promoter-binding protein	HKSDNETNLQQQVVWGNR	S3(Phospho)
Q7Z401	C-myc promoter-binding protein	TPSIDLQR	S3(Phospho)
A6NC98	Coiled-coil domain-containing protein 88B	SLEPPPGSPGEAPLAGAAPSLQDEVR	S8(Phospho)
P21127	Cyclin-dependent kinase 11B	EYGSPLKAYTPVVVTLWYR	S4(Phospho); T10(Phospho)
P27707	Deoxycytidine kinase	WCNVQSTQDEFEELTMSQK	C2(Carbamidomethyl); S17(Phospho)
Q02880	DNA topoisomerase 2-beta	KTSFDQDSDVDIFPSDFPTEPPSLPR	T2(Phospho)
P85037	Forkhead box protein K1	SGGLQTPECLSR	C9(Carbamidomethyl); S11(Phospho)
Q32MZ4	Leucine-rich repeat flightless-interacting protein 1	IDGATQSSPAEPK	S8(Phospho)
Q8N1G4	Leucine-rich repeat-containing protein 47	YTLENKEEGSLSDTEADAVSGQLPDPTTNPSAGK	S12(Phospho)
Q8NHL6	Leukocyte immunoglobulin-like receptor subfamily B member 1	REMASPPSPLSGEFLDTK	M3(Oxidation); S8(Phospho)
Q8NHJ6	Leukocyte immunoglobulin-like receptor subfamily B member 4	EMASPPSPLSGEFLDTK	S7(Phospho)
Q8NHJ6	Leukocyte immunoglobulin-like receptor subfamily B member 4	REMASPPSPLSGEFLDTK	M3(Oxidation); S8(Phospho)
Q8NHJ6	Leukocyte immunoglobulin-like receptor subfamily B member 4	RSSPAADVQGENFCAAVK	S2(Phospho); C14(Carbamidomethyl)
Q9GZY6	Linker for activation of T-cells family member 2	HGSEEAYIDPIAMEYYNWGR	Y7(Phospho); M13(Oxidation)
P22897	Macrophage mannose receptor 1	VHLPQEGAFENTLYFNSQSSPGTSDMK	S20(Phospho)
Q5VSK2	Macrophage mannose receptor 1-like protein 1	VHLPQEGAFENTLYFNSQSSPGTSDMK	S20(Phospho)
Q13459	Myosin-IXb	RTSFSTSDVSK	S3(Phospho)
Q13615	Myotubularin-related protein 3	TRSYDNLTTACDNTVPLASR	S3(Phospho); C11(Carbamidomethyl)
P29966	Myristoylated alanine-rich C-kinase substrate	EAPAEGEAAEPGSPTAAEGEAASAASSTSSPK	T15(Phospho)
Q09666	Neuroblast differentiation-associated protein AHNAK	VSMPDVELNLKSPK	S12(Phospho)
Q09666	Neuroblast differentiation-associated protein AHNAK	GHYEVTGSDDETGKLQGSGVSLASK	T6(Phospho)
Q09666	Neuroblast differentiation-associated protein AHNAK	ISMSEVDLNVAAPK	S2(Phospho)
P67809	Nuclease-sensitive element-binding protein 1	SVGDGETVEFDVVEGEK	S1(Phospho)
P06748	Nucleophosmin	DELHIVEAEAMNYEGSPIK	S16(Phospho)
Q15154	Pericentriolar material 1 protein	NRHSAQTEEPVQAK	S4(Phospho)
Q92835	Phosphatidylinositol-3,4,5-trisphosphate 5-phosphatase 1	GESPPTPPGQPPISPK	T6(Phospho); S14(Phospho)
Q15149	Plectin	GYYSPYSVSGSGSTAGSR	S17(Phospho)
Q8NDX5	Polyhomeotic-like protein 3	MDRTPPPPTLSPAAITVGR	T4(Phospho); S11(Phospho)
Q15185	Prostaglandin E synthase 3	DWEDDSDEDMSNFDR	S6(Phospho)
Q96E09	Protein FAM122A	RIDFIPVSPAPSPTR	S12(Phospho)
Q96ST2	Protein IWS1 homolog	HQASDSENEELPKPR	S4(Phospho); S6(Phospho)
Q96ST2	Protein IWS1 homolog	HQASDSENEEPPKPR	S6(Phospho)
Q8N163	Protein KIAA1967	SVASNQSEMEFSSLQDMPK	S4(Phospho)
Q9ULU4	Protein kinase C-binding protein 1	TGQAGSLSGSPKPFSPQLSAPITTK	S10(Phospho)
Q8ND56	Protein LSM14 homolog A	SPTMEQAVQTASAHLPAPAAVGR	T3(Phospho)
Q92597	Protein NDRG1	SHTSEGAHLDITPNSGAAGNSAGPK	S1(Phospho)
O15258	Protein RER1	VDPSLMEDSDDGPSLPTK	S9(Phospho)
O15027	Protein transport protein Sec16A	AQQELVPPQQQASPPQLPK	S13(Phospho)
O95487	Protein transport protein Sec24B	DSRPLSPILHIVK	S6(Phospho)
Q14242	P-selectin glycoprotein ligand 1	NYSPTEMVCISSLLPDGGEGPSATANGGLSK	S3(Phospho); C9(Carbamidomethyl)
Q14671	Pumilio homolog 1	RPGQSFHVNSEVNSVLSPR	S17(Phospho)
P08559	Pyruvate dehydrogenase E1 component subunit alpha, somatic form, mitochondrial	YGMGTSVER	S6(Phospho)
Q9H6Z4	Ran-binding protein 3	TSSLTQFPPSQSEER	S3(Phospho)
P61006	Ras-related protein Rab-8A	KLEGNSPQGSNQGVK	S10(Phospho)
O43566	Regulator of G-protein signaling 14	MVLAVSDGELSSTTGPQGQGEGR	S6(Phospho)
P49795	Regulator of G-protein signaling 19	LQPLPSCEVCATPSPEEVQSWAQSFDK	S6(Phospho); C7(Carbamidomethyl); C10(Carbamidomethyl)
P42331	Rho GTPase-activating protein 25	GDTLASPNSETGPGK	S6(Phospho)
Q7Z6I6	Rho GTPase-activating protein 30	SYAFETQANPGK	Y2(Phospho)
Q15052	Rho guanine nucleotide exchange factor 6	KDSIPQVLLPEEEKLIIEETR	S3(Phospho)
Q15052	Rho guanine nucleotide exchange factor 6	MSGFIYQGK	S2(Phospho)
Q14155	Rho guanine nucleotide exchange factor 7	MSGFIYQGK	S2(Phospho)
Q15418	Ribosomal protein S6 kinase alpha-1	KAYSFCGTVEYMAPEVVNR	C6(Carbamidomethyl); T8(Phospho)
P51812	Ribosomal protein S6 kinase alpha-3	KAYSFCGTVEYMAPEVVNR	C6(Carbamidomethyl); T8(Phospho)
P51812	Ribosomal protein S6 kinase alpha-3	NSIQFTDGYEVK	S2(Phospho)
Q9UK32	Ribosomal protein S6 kinase alpha-6	KAYSFCGTVEYMAPEVVNR	C6(Carbamidomethyl); T8(Phospho)
Q6PD62	RNA polymerase-associated protein CTR9 homolog	GEEGSDDDETENGPKPK	S5(Phospho)
Q9Y3Z3	SAM domain and HD domain-containing protein 1	EQIVGPLESPVEDSLWPYK	S9(Phospho)
Q9UHR5	SAP30-binding protein	GGLVSDAYGEDDFSR	S14(Phospho)
Q9UQ35	Serine/arginine repetitive matrix protein 2	AGMSSNQSISSPVLDAVPR	S11(Phospho)
Q9UQ35	Serine/arginine repetitive matrix protein 2	SSSPVTELASR	S2(Phospho)
Q9UQ35	Serine/arginine repetitive matrix protein 2	GEFSASPMLK	S6(Phospho); M8(Oxidation)
Q9UQ35	Serine/arginine repetitive matrix protein 2	SSGHSSSELSPDAVEK	S10(Phospho)
Q8TD19	Serine/threonine-protein kinase Nek9	HCDSINSDFGSESGGCGDSSPGPSASQGPR	C2(Carbamidomethyl); C16(Carbamidomethyl); S20(Phospho)
Q13177	Serine/threonine-protein kinase PAK 2	DGFPSGTPALNAK	S5(Phospho)
Q9BXS9	Solute carrier family 26 member 6	GASVSINVNTSLEDMR	S11(Phospho)
P28290	Sperm-specific antigen 2	TPLGASLDEQSSSTLK	S6(Phospho)
Q9H7N4	Splicing factor, arginine/serine-rich 19	FDIYDPFHPTDEAYSPPPAPEQK	S15(Phospho)
Q9H2G2	STE20-like serine/threonine-protein kinase	RDSFIGTPYWMAPEVVMCETSK	S3(Phospho); M11(Oxidation); M17(Oxidation); C18(Carbamidomethyl)
P31948	Stress-induced-phosphoprotein 1	ALSVGNIDDALQCYSEAIK	S3(Phospho); C13(Carbamidomethyl)
O00161	Synaptosomal-associated protein 23	EDEMEENLTQVGSILGNLK	M4(Oxidation); S13(Phospho)
Q9UMZ2	Synergin gamma	ETSFGSSENITMTSLSK	S6(Phospho)
Q92609	TBC1 domain family member 5	NISSSPSVESLPGGR	S7(Phospho)
Q9Y2W1	Thyroid hormone receptor-associated protein 3	GSFSDTGLGDGK	S4(Phospho)
Q9Y2W1	Thyroid hormone receptor-associated protein 3	IDISPSTFR	S4(Phospho)
Q9Y2W1	Thyroid hormone receptor-associated protein 3	KSPVGKSPPSTGSTYGSSQK	S7(Phospho)
Q13263	Transcription intermediary factor 1-beta	SRSGEGEVSGLMR	S3(Phospho); M12(Oxidation)
Q6UWF3	Transmembrane protein C17orf87	MYENVLNESPVQLPPLPPR	S9(Phospho)
O75643	U5 small nuclear ribonucleoprotein 200 kDa helicase	EEASDDDMEGDEAVVR	S4(Phospho)
P54578	Ubiquitin carboxyl-terminal hydrolase 14	ASGEMASAQYITAALR	S2(Phospho)
O94888	UBX domain-containing protein 7	SESLIDASEDSQLEAAIR	S1(Phospho); S11(Phospho)
Q96A57	UPF0414 transmembrane protein C20orf30	YSRLSSTDDGYIDLQFK	S5(Phospho); S6(Phospho)
Q9Y4E1	WASH complex subunit FAM21C	KVQSTADIFGDEEGDLFK	S4(Phospho)
Q7Z3T8	Zinc finger FYVE domain-containing protein 16	NEIIQSPISQVPSVEK	S6(Phospho); S13(Phospho)
O95218	Zinc finger Ran-binding domain-containing protein 2	ENVEYIEREESDGEYDEFGR	S11(Phospho)
